# Supplementary material for: Emergency admissions and long-term conditions during transition from paediatric to adult care: a cross-sectional study using Hospital Episode Statistics data
Source: BMJ Open. 2018 Jun 22;8(6):e021015. doi: 10.1136/bmjopen-2017-021015 (PMC6020943; doi:10.1136/bmjopen-2017-021015)
Supplement: Supplementary file 1 [file bmjopen-2017-021015supp001.pdf]

Appendix A: Code lists

In order to identify primary diagnoses groups in Table 2 and Figures in Appendix C, we used the following ICD-10 codes:

Table A1: ICD-10 codes used to define condition groups

Primary diagnoses group	ICD-10 codes
Long-term conditions	
Diabetes	E10-E14, G59.0, G63.2, I79.2, M14.2, N08.3, O24.0, O24.1, O24.2, O24.3, Y42.3
Asthma and chronic lower respiratory disease	J41-J47
Epilepsy	F80.3, G40.0, G40.1, G40.2, G40.3, G40.4, G40.6, G40.7, G41, G40.8, G40.9, R56.8, Y46.0, Y46.1, Y46.2, Y46.3, Y46.4, Y46.5, Y46.6
Non-infective enteritis and colitis / inflammatory bowel disease	K50, K51, K52
Mental health conditions	
Substance use	F10-F19
Schizophrenia, schizotypical and delusional disorders	F20-F29
Personality disorders	F60, F61, F69
Mood disorders	F30-F39
Infections	
Enteric / gastrointestinal infection	A00-A09, I88.0, K23.0, K23.1, K25-K28, K29.3, K29.4, K29.5, K35-K37, K52.8, K52.9, K61, K63.0, K63.2, K65.0, K67.8, K90.8, K93.0, R11
Respiratory infection	A15-A19, A48.1, A48.2, B59, J00-J06, J10-J22, J32, J36, J37, J39.0, J39.1, J40-J42, J43.0, J44.0, J47, J56, J85, J86, J98.8, N74.0, N74.1
Genitourinary infection	N30.0, N34.1, N35.1, N37, N39.0, N41.0, N41.1, N41.2, N41.3, N43.1, N45, N48.1, N48.2, N49, N51, N70-N74, N75.1, N76.4, N87
Symptoms and signs	
Abdominal pain	R10-R19, K59
General symptoms and signs	R50-R69
Respiratory and circulatory symptoms	R00-R09
Other symptoms	R20-49, R70-R99
Other conditions	
Non-inflammatory disease of the female genitourinary system	N80-N98
Other joint/soft tissue disorders	M20-M25, M70-M79
Diseases of male genital organs	N40-N51

Codes for long-term conditions (apart from sickle cell disease and inflammatory bowel disease) were taken from *Hardelid et al.*[1], codes for infections were taken from *Baker et al.*[2]. Other groups are based on ICD-10 subchapters.

1. Hardelid P, Dattani N, Davey J, et al. Overview of child deaths in the four UK countries. HQIP bit.ly/hardelidreport

2. Barker MG, Barnard LT, Kvalsvig A, et al. Increasing incidence of serious infectious diseases and inequalities in New Zealand: a national epidemiological study. *The Lancet* 2012;**379**:1112-9.
