# Supplementary material for: Emergency admissions and long-term conditions during transition from paediatric to adult care: a cross-sectional study using Hospital Episode Statistics data
Source: BMJ Open. 2018 Jun 22;8(6):e021015. doi: 10.1136/bmjopen-2017-021015 (PMC6020943; doi:10.1136/bmjopen-2017-021015)
Supplement: Supplementary file 2 [file bmjopen-2017-021015supp002.pdf]

Appendix B: additional tables and figures

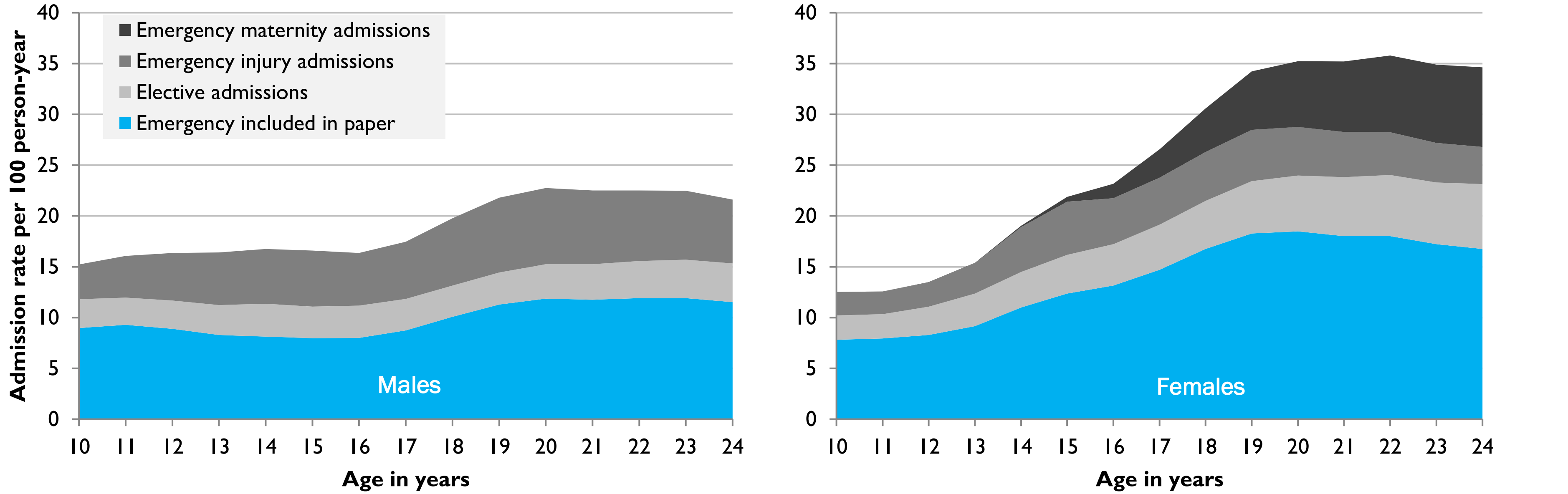

Figure B1: Emergency admission included in context: emergency admissions included in the primary analyses are shown in blue, while excluded admissions (elective admissions, emergency injury and maternity admissions) are shown in grey.

**Table B1:** Baseline characteristics of included admissions			
	**Overall**	**No LTC**	**LTC**
**Covariate**	**Number of admissions (%)**		
**Total**	**1,109,978**	**410,144**	**699,834**
Before transition (10-15 years)	327,531 (29.5)	142,206 (34.7)	185,325 (26.5)
During transition (16-18 years)	224,722 (20.3)	81,676 (19.9)	143,046 (20.4)
After transition (19-24 years)	557,725 (50.3)	186,262 (45.4)	371,463 (53.1)
Females	644,994 (58.1)	229,651 (56.0)	415,343 (59.4)
IMD '04			
1 - Most deprived	319,837 (28.8)	108,211 (26.4)	211,626 (30.2)
2	242,428 (21.8)	85,930 (21.0)	156,498 (22.4)
3	198,237 (17.9)	74,583 (18.2)	123,690 (17.7)
4	172,479 (15.5)	68,364 (16.7)	104,115 (14.9)
5 – Least deprived	159,588 (14.4)	65,817 (16.1)	93,771 (13.4)
Missing	17,409 (1.6)	7,239 (1.8)	10,134 (1.5)
Ethnicity			
White	860,552 (77.5)	307,092 (74.9)	553,460 (79.1)
Black or Black British	45,564 (4.1)	14,093 (3.4)	31,471 (4.5)
Asian or Asian British	75,393 (6.8)	30,460 (7.4)	44,933 (6.4)
Mixed	44,863 (4.0)	17,414 (4.3)	27,449 (3.9)
Unknown	20,251 (1.8)	10,118 (2.5)	10,133 (1.5)
Missing	63,355 (5.7)	30,967 (7.6)	32,388 (4.6)

Table B2: Admission rates, incidence rate ratios and trends in rates before, during, and after transition to adult health care for children with and without underlying long-term conditions (LTCs)

	Before transition		After transition		Before	During	After
	Percentage of admissions 10-15 years	Average rate 10-15 years	Average rate 19-24 years		Trend age 10-15 years	Trend age 16-18 years	Trend age 19-25 years
**FEMALES**		Rate per 1,000 person-years		IRR (99% CI)†	Annual change in admission rate per 1,000 person-years		
**Overall***	**100**	**32.5**	**52.7**	**1.62 (1.61 – 1.63)**	**↑4.48**	**↑3.78**	**↓-1.39**
No LTCs	41.8	13.6	17.1	1.26 (1.25 – 1.28)	↗0.87	↗0.92	↘-0.61
History of LTCs†	58.2	18.9	35.5	1.88 (1.87 – 1.89)	↑3.61	↑2.86	↘-0.78
**LTC groups**							
Mental health disorders	20.5	6.7	17.8	2.67 (2.65 – 2.68)	↑2.87	↑1.48	↘-0.63
Substance use	2.9	0.9	4.6	4.94 (4.90 – 4.98)	↗0.51	↗0.77	→-0.09
Cancer/blood disorders	6.8	2.2	2.6	1.20 (1.17 – 1.23)	→0.09	→-0.02	→0.00
Chronic infections	0.4	0.1	0.3	2.34 (2.22 – 2.45)	→0.02	→0.02	→0.01
Respiratory disorders	16.3	5.3	10.3	1.94 (1.92 – 1.96)	↗0.53	↑1.11	↘-0.23
Asthma	11.4	3.7	6.6	1.79 (1.77 – 1.81)	↗0.29	↗0.79	↘-0.20
MEDReG‡ disorders	18.8	6.1	16.2	2.66 (2.64 – 2.67)	↗0.66	↑2.12	↗0.15
Inflammatory bowel disease	3.2	1.0	2.4	2.35 (2.31 – 2.39)	→0.05	↗0.30	→0.04
Diabetes	5.2	1.7	2.0	1.16 (1.13 – 1.20)	↗0.19	↗0.13	↘-0.15
Musculoskeletal/skin disorders	7.7	2.5	5.3	2.11 (2.09 – 2.14)	↗0.33	↗0.40	→0.05
Neurological disorders	13.1	4.3	6.5	1.52 (1.50 – 1.54)	↗0.44	↗0.23	→0.06
Epilepsy	5.2	1.7	1.9	1.13 (1.09 – 1.21)	↗0.11	→-0.02	→-0.04
Cardiovascular disorders	2.1	0.7	0.8	1.16 (1.10 – 1.21)	→0.05	→-0.07	→-0.01
Multiple LTCs	17.8	5.8	15.2	2.62 (2.61 – 2.64)	↑1.00	↑1.66	→-0.05
**MALES**					Annual change in admission rate per 1,000 person-years		
**Overall***	**100**	**27.5**	**34.6**	**1.26 (1.25 – 1.27)**	**↘-0.70**	**↑2.80**	**→0.07**
No LTCs	45.3	12.4	12.0	0.96 (0.95 – 0.98)	↘-0.97	↗0.88	↘-0.23

History of LTCs†	54.7	15.0	22.6	1.50 (1.49 – 1.51)	↗0.27	↑1.91	↗0.30
**LTC groups**							
Mental health disorders	13.4	3.7	12.6	3.43 (3.41 – 3.45)	↗0.61	↑1.75	↗0.30
Substance use	2.0	0.5	4.9	9.01 (8.97 – 9.06)	↗0.29	↗0.98	↗0.22
Cancer/blood disorders	8.8	2.4	2.2	0.92 (0.89 – 0.96)	→0.02	→-0.07	→0.02
Chronic infections	0.5	0.1	0.5	2.44 (2.34 – 2.55)	→0.01	→0.02	→0.05
Respiratory disorders	19.0	5.2	4.9	0.94 (0.92 – 0.96)	↘-0.33	↗0.17	→-0.08
Asthma	13.8	3.8	3.1	0.82 (0.79 – 0.84)	↘-0.36	↗0.14	→-0.09
MEDReG‡ disorders	19.1	5.2	7.9	1.51 (1.49 – 1.53)	↗0.11	↗0.54	↗0.28
Inflammatory bowel disease	4.2	1.1	1.9	1.62 (1.58 – 1.66)	→-0.03	↗0.18	→0.05
Diabetes	4.7	1.3	1.4	1.08 (1.04 – 1.12)	↗0.11	↗0.16	→-0.06
Musculoskeletal/skin disorders	6.8	1.9	2.6	1.38 (1.35 – 1.41)	→0.08	→0.08	→0.10
Neurological disorders	14.5	4.0	4.1	1.04 (1.02 – 1.06)	→0.00	→-0.03	→0.07
Epilepsy	5.9	1.6	1.8	1.14 (1.10 – 1.17)	→-0.04	→0.00	→0.02
Cardiovascular disorders	2.6	0.7	0.7	0.94 (0.88 – 0.99)	→-0.02	→-0.06	→0.02
Multiple LTCs	19.0	5.2	8.5	1.63 (1.61 – 1.64)	↗0.19	↗0.55	↗0.23

*Excluding admissions for injury or maternity; †At least 1 admission with a record of a long-term condition in the previous 5 years (including current admission); ‡Metabolic, endrocrine, digestive, renal or genitourinary disorders

†Rate ratio comparing emergency admission rates before and after transition

Trends:

↑ increasing by ≥1.0 admission per 1,000 person-year (py);

↗ increasing by 0.1-0.99 per 1,000py;

→ stable at -0.1 to 0.1 per 1,000 py;

↘ decreasing by -0.1 to -0.99 per 1,000py;

↓ decreasing by ≤-1.0 per 1,000py

Table B3: Admission rates, incidence rate ratios and trends in rates before, during, and after transition to adult health care by primary diagnosis

	Proportion of admissions 10-15 years	Average rate 10-15 years	Average rate 19-24 years	IRR (99% CI)†	Trend age 10-15 years	Trend age 16-18 years	Trend age 19-25 years
**FEMALES***	Rate per 1,000 person-years				Annual change in admission rate per 1,000 person-years		
**Long-term conditions**							
Any long-term condition	26.4	9.8	19.8	2.02 (2.00 – 2.03)	↑1.04	↑2.54	↘-0.12
Diabetes	3.7	1.2	1.4	1.18 (1.13 – 1.22)	→0.10	↗0.26	↘-0.22
Asthma	4.5	1.4	1.6	1.17 (1.13 – 1.20)	↘-0.11	↗0.14	→-0.02
Inflammatory bowel disease	2.3	0.7	2.1	2.93 (2.88 – 2.98)	→0.03	↗0.37	→0.03
Epilepsy	3.4	1.1	1.2	1.09 (1.05 – 1.14)	→0.07	→0.02	→-0.06
**Infection**							
Any infection	25.4	8.4	14.4	1.72 (1.70 – 1.73)	↗0.26	↑1.66	↘-0.50
Enteric/GI infection	9.4	2.9	4.1	1.40 (1.38 – 1.43)	→0.06	↗0.28	↘-0.11
Respiratory tract infection	7.0	2.2	3.1	1.45 (1.42 – 1.48)	→0.05	↗0.30	↘-0.13
Genitourinary infection	2.5	0.8	3.2	4.10 (4.05 – 4.14)	↗0.11	↗0.58	↘-0.14
**Symptoms**							
Any symptoms	35.8	11.3	20.5	1.83 (1.81 – 1.84)	↑1.31	↑1.95	↘-0.48
Abdominal pain	22.6	7.1	12.5	1.76 (1.75 – 1.78)	↗0.79	↑1.26	↘-0.46
General symptoms	6.9	2.2	3.4	1.58 (1.55 – 1.61)	↗0.31	↗0.12	→-0.04
**Other**							
Any other	12.4	2.6	4.9	1.87 (1.85 – 1.90)	↗0.33	↗0.44	→0.06
Non-inflammatory disease of the female GT	2.3	0.6	1.8	3.10 (3.04 – 3.15)	↗0.22	↗0.20	→-0.04
Other joint/soft tissue disorders	2.4	0.5	0.5	0.95 (0.89 – 1.02)	→-0.01	→0.04	→0.00
**MALES***					Annual change in admission rate per 1,000 person-years		

**Long-term conditions**							
Any long-term condition	25.8	8.9	14.4	1.61 (1.60 – 1.62)	↗0.17	↑1.50	↗0.29
Diabetes	3.3	0.9	1.1	1.17 (1.12 – 1.22)	→0.09	↗0.21	↘-0.13
Asthma	5.5	1.5	0.8	0.49 (0.44 – 0.53)	↘-0.29	→-0.05	→-0.03
Inflammatory bowel disease	2.9	0.7	2.0	2.95 (2.91 – 3.00)	→0.02	↗0.24	→0.01
Epilepsy	4.1	1.2	1.2	1.01 (0.97 – 1.06)	→-0.01	→0.05	→-0.01
**Infection**							
Any infection	28.6	8.5	10.9	1.29 (1.27 – 1.30)	↘-0.47	↑1.08	↘-0.14
Enteric/GI infection	12.7	3.6	3.9	1.08 (1.06 – 1.11)	↘-0.15	↗0.15	→-0.06
Respiratory tract infection	7.0	2.0	2.7	1.38 (1.35 – 1.41)	↘-0.13	↗0.42	↘-0.11
Genitourinary infection	1.7	0.5	0.8	1.63 (1.57 – 1.69)	→-0.03	↗0.11	→-0.01
**Symptoms**							
Any symptoms	29.2	8.3	10.1	1.21 (1.20 – 1.23)	↘-0.52	↗0.97	→0.05
Abdominal pain	15.4	4.4	4.0	0.90 (0.88 – 0.92)	↘-0.54	↗0.28	→-0.02
General symptoms	6.8	1.9	2.3	1.20 (1.17 – 1.24)	→0.02	↗0.19	→-0.01
**Other**							
Any other	16.4	3.4	3.7	1.10 (1.08 – 1.13)	↘-0.21	↗0.26	→0.02
Diseases of male genital organs	6.0	1.2	0.6	0.49 (0.44 – 0.55)	→-0.08	→-0.03	→-0.04
Other joint/soft tissue disorders	2.3	0.5	0.4	0.94 (0.87 – 1.01)	→-0.06	→0.00	→0.02

*Excluding admissions for injury or maternity

†Incidence rate ratio comparing emergency admission rates before and after transition

Trends:

↑ increasing by ≥1.0 admission per 1,000 person-year (py);

↗ increasing by 0.1-0.99 per 1,000py;

→ stable at -0.1 to 0.1 per 1,000 py;

↘ decreasing by -0.1 to -0.99 per 1,000py;

↓ decreasing by ≤-1.0 per 1,000py

Table B4: Most frequent diagnoses for CYP with and without underlying LTCs

Rank	CYP with LTCs 10-15 years	CYP with LTCs 16-18 years	CYP with LTCs 19-24 years	CYP without LTCs 10-15 years	CYP without LTCs 16-18 years	CYP without LTCs 19-24 years
	Diagnosis group* (frequency, % of admissions)			Diagnosis group* (frequency, % of admissions)		
1	Asthma (13,209, 7.1%)	Abdominal and pelvic pain (14,643, 10.2%)	Abdominal and pelvic pain (41,809, 11.3%)	Abdominal and pelvic pain (34,348, 24.2%)	Abdominal and pelvic pain (19,231, 23.6%)	Abdominal and pelvic pain (39,952, 21.5%)
2	Abdominal and pelvic pain (13,015, 7.0%)	Poisoning by nonopioid analgesics, antipyretics and antirheumatics (12,835, 9.0%)	Poisoning by nonopioid analgesics, antipyretics and antirheumatics (20,632, 5.6%)	Acute appendicitis (11,699, 8.2%)	Acute appendicitis (6,266, 7.7%)	Pain in throat and chest (10,574, 5.7%)
3	Insulin-dependent diabetes mellitus (10,277, 5.6%)	Insulin-dependent diabetes mellitus (6,694, 4.7%)	Pain in throat and chest (10,737, 2.9%)	Other functional intestinal disorders (4,783, 3.4%)	Acute tonsillitis (4,272, 5.2%)	Acute appendicitis (10,206, 5.5%)
4	Poisoning by nonopioid analgesics, antipyretics and antirheumatics (10,105, 5.5%)	Asthma (4,191, 2.9%)	Other noninfective gastroenteritis and colitis (10,546, 2.8%)	Viral infection of unspecified site (4,615, 3.3%)	Pain in throat and chest (3,105, 3.8%)	Headache (6,984, 3.8%)
5	Epilepsy (6,146, 3.3%)	Mental and behavioral disorders due to use of alcohol (3,796, 2.7%)	Insulin-dependent diabetes mellitus (9,971, 2.7%)	Acute tonsillitis (4,275, 3.0%)	Other disorders of urinary system (3,046, 3.7%)	Other disorders of urinary system (6,029, 3.2%)

*Diagnoses are grouped by 3-character ICD codes (e.g. E84 for cystic fibrosis)
Signs and symptoms are highlighted

Table B5: Most frequent diagnoses for long (10+ days) lengths of stay by age group and gender

Rank	**Males**			**Females**		
	**10-15 years**	**16-18 years**	**19-24 years**	**10-15 years**	**16-18 years**	**19-24 years**
	Diagnosis group* (frequency, *average length of stay*)			Diagnosis group* (frequency, *average length of stay*)		
1	Cystic Fibrosis (414, *8.6*)	Unknown and unspecified causes of morbidity (354, ***53.4***)	Schizophrenia (3,268, ***140.0***)	Cystic fibrosis (612, *8.6*)	Cystic fibrosis (390, *8.8*)	Abdominal and pelvic pain (1,111, *2.0*)
2	Pneumonia, organism unspecified (205, *4.6*)	Pneumothorax (313, *6.0*)	Unknown and unspecified causes of morbidity (2,196, ***97.6***)	Eating disorders (519, ***41.9***)	Abdominal and pelvic pain (386, *1.8*)	Unknown and unspecified causes of morbidity (1,096, ***40.5***)
3	Lymphoid leukaemia (193, *4.9*)	Schizophrenia (268, ***119.7***)	Acute and transient psychotic disorders (773, ***44.3***)	Pneumonia, organism unspecified (221, *4.6*)	Unknown and unspecified causes of morbidity (328, ***53.4***)	Specific personality disorders (917, ***32.5***)
4	Osteomyelitis (175, ***11.0***)	Cystic Fibrosis (254, *8.8*)	Crohn disease (648, *7.4*)	Lymphoid leukaemia (164, *4.9*)	Eating disorders (316, ***49.5***)	Cystic fibrosis (761, ***11.4***)
5	Unspecified acute lower respiratory infection / Acute appendicitis (171, *3.2 / 3.1*)	Ulcerative colitis (240, *8.9*)	Ulcerative colitis (590, *8.7*)	Unspecified acute lower respiratory infection (160, *3.2*)	Crohn disease (211, *7.0*)	Crohn disease (721, *7.4*)

*Diagnoses are grouped by 3-character ICD codes (e.g. E84 for cystic fibrosis)
Average lengths of stay are calculated for all emergency admissions (excluding injury and maternity-related admissions). Average lengths of stay of 10 days or greater are shown in bold.
Mental health conditions are highlighted in blue, unspecified conditions and conditions that could represent psychosomatic symptoms are highlighted in yellow.
