# Supplementary material for: Emergency admissions and long-term conditions during transition from paediatric to adult care: a cross-sectional study using Hospital Episode Statistics data
Source: BMJ Open. 2018 Jun 22;8(6):e021015. doi: 10.1136/bmjopen-2017-021015 (PMC6020943; doi:10.1136/bmjopen-2017-021015)
Supplement: Supplementary file 3 [file bmjopen-2017-021015supp003.pdf]

Appendix C: Sensitivity analyses

In Figure A1 in Appendix A, we showed the overall admission rate for children and young people aged 10-24 years old. Here we show the results, as they would have been in Table 1, if we had included all emergency admission, compared to the results presented in the paper (excluding injury admissions, and maternity/pregnancy related emergency admissions for females, Table C1).

Table C1: Sensitivity analysis of exclusion criteria on rates and trends in emergency admission rates before, during and transition from pediatric to adult care

	Proportion of admissions 10-15 years	Average rate 10-15 years	Average rate 19-24 years		Trend age 10-15 years	Trend age 16-18 years	Trend age 19-25 years
**FEMALES**	Rate per 1,000 person-years			IRR† (99% CI)	Annual change in admission rate per 1,000 py		
Overall	*100*	39.4	81.6	2.07 (2.06 – 2.08)	↑3.61	↑2.86	↘-0.78
Overall, excluding injury/maternity admissions	82.4	32.5	52.7	1.62 (1.61 – 1.63)	↑4.48	↑3.78	↓-1.39
Injury admissions	16.7	6.6	6.9	1.05 (1.03-1.06)	→0.10	↗0.41	↘-0.31
Maternity/pregnancy admissions	0.9	0.4	22.2	61.9 (61.8 – 62.0	↗0.26	↑4.62	↑1.34
**MALES**							
Overall	*100*	41.0	50.8	1.24 (1.23 – 1.25)	↗0.27	↑1.91	↗0.30
Overall, excluding injury admissions	67.0	27.5	34.6	1.26 (1.25 – 1.27)	↘-0.70	↑2.80	→0.07
Injury admissions	33.0	13.5	16.2	1.20 (1.19 – 1.21)	↗0.94	↑1.49	↘-0.52

*Excluding maternity/pregnancy related admissions and injury admissions
†Incidence rate ratio comparing emergency admission rates before and after transition

By excluding injury and maternity/pregnancy related emergency admissions, we have slightly underestimated the increase in emergency admission rates across transition for both males and females.

In the manuscript, we assessed children with underlying long-term conditions as a separate group. We assessed whether children and young people had a code indicating a LTC recorded in any admission record (emergency or elective) in the previous 5 years. As a sensitivity analysis, we explored the effects of only using admission records from the last year or last 3 years (Table C2).

Table C2: Sensitivity analysis of the effect of including long-term conditions (LTCs) recorded in the last year, last 3 years, or last 5 years

	Proportion of admissions 10-15 years	Average rate 10-15 years	Average rate 19-24 years		Trend age 10-15 years	Trend age 16-18 years	Trend age 19-25 years
**FEMALES***	Rate per 1,000 person-years			IRR[†] (99% CI)	Annual change in admission rate per 1,000 py		
No LTCs	41.8	13.6	17.1	1.26 (1.25 – 1.28)	↗0.87	↗0.92	↘-0.61
LTC recorded in previous year	56.0	18.2	33.3	1.83 (1.82-1.84)	↑3.49	↑2.54	↘-0.77
LTC recorded in previous 3 years	57.5	18.7	34.7	1.86 (1.85-1.87)	↑3.58	↑2.71	↘-0.79
LTC recorded in previous 5 years	58.2	18.9	35.5	1.88 (1.87-1.89)	↑3.61	↑2.86	↘-0.78
**MALES***							
No LTCs	45.3	12.4	12.0	0.96 (0.95-0.98)	↘-0.97	↗0.88	↘-0.23
LTC recorded in previous year	52.6	14.4	21.7	1.50 (1.49 – 1.51)	↗0.30	↑1.84	↗0.26
LTC recorded in previous 3 years	55.8	15.3	22.3	1.45 (1.44 – 1.47)	↗0.29	↑1.90	↗0.28
LTC recorded in previous 5 years	56.0	15.4	22.6	1.47 (1.46 – 1.48)	↗0.27	↑1.91	↗0.30

*Excluding maternity/pregnancy related admissions and injury admissions
†Incidence rate ratio comparing emergency admission rates before and after transition

When using records for the previous five years, we find slightly higher emergency admission rates for children with underlying long-term conditions compared to looking back just one or three years. However, the relatively small differences indicate most LTCs were recorded in the year prior to the index emergency admission.
