# Supplementary material for: Emergency admissions and long-term conditions during transition from paediatric to adult care: a cross-sectional study using Hospital Episode Statistics data
Source: BMJ Open. 2018 Jun 22;8(6):e021015. doi: 10.1136/bmjopen-2017-021015 (PMC6020943; doi:10.1136/bmjopen-2017-021015)
Supplement: Author's manuscript [file bmjopen-2017-021015.draft_revisions.pdf]

BMJ Open

BMJ Open is committed to open peer review. As part of this commitment we make the peer review history of every article we publish publicly available.

When an article is published we post the peer reviewers' comments and the authors' responses online. We also post the versions of the paper that were used during peer review. These are the versions that the peer review comments apply to.

The versions of the paper that follow are the versions that were submitted during the peer review process. They are not the versions of record or the final published versions. They should not be cited or distributed as the published version of this manuscript.

BMJ Open is an open access journal and the full, final, typeset and author-corrected version of record of the manuscript is available on our site with no access controls, subscription charges or pay-per-view fees (http://bmjopen.bmj.com).

If you have any questions on BMJ Open's open peer review process please email info.bmjopen@bmj.com

BMJ Open

Emergency admissions and long-term conditions during transition from paediatric to adult care: a cross-sectional study using Hospital Episode Statistics data

Journal:	*BMJ Open*
Manuscript ID	bmjopen-2017-021015
Article Type:	Research
Date Submitted by the Author:	08-Dec-2017
Complete List of Authors:	Wijlaars, Linda; UCL, Population, Policy and Practice; University College London, Administrative Data Research Centre for England<br>Hardelid, Pia; UCL Institute of Child Health, Centre for Paediatric Epidemiology and Biostatistics<br>Guttmann, Astrid; Institute for Clinical Evaluative Sciences, ; The Hospital for Sick Children, Division of Pediatric Medicine<br>Gilbert, Ruth; UCL Institute of Child Health, Centre for Paediatric Epidemiology and Biostatistics
Keywords:	PAEDIATRICS, Chronic conditions, Administrative hospital data, MENTAL HEALTH, Emergency admissions, Transition from paediatric to adult care

SCHOLARONE™
Manuscripts

For peer review only

Emergency admissions and long-term conditions during transition from paediatric to adult care: a cross-sectional study using Hospital Episode Statistics data

Linda PMM Wijlaars [a,b] (linda.wijlaars@ucl.ac.uk), Pia Hardelid[a] (p.hardelid@ucl.ac.uk), Astrid Guttmann[c] (astrid.guttmann@ices.on.ca), Ruth Gilbert[a,b] (r.gilbert@ucl.ac.uk)

Affiliations:

[a]Population, Policy and Practice / Children's Policy Research Unit, University College London Great Ormond Street Institute of Child Health, 30 Guilford Street, WC1X 9ED London, UK;

[b]Administrative Data Research Centre for England, University College London, 222 Euston Road, NW1 2DA London, United Kingdom,

[c]Institute of Clinical Evaluation Sciences, Toronto, Ontario, Canada

Corresponding author: Linda Wijlaars

Short title: Emergency admissions during transition

Keywords: Administrative data, transition, paediatrics, chronic conditions, mental health, hospitalizations

Abstract word count: 274

Article word count: 3185

For peer review only

Abstract:

Objectives: To determine whether changes in emergency admission rates during transition from paediatric to adult hospital services differed in children and young people (CYP) with and without underlying long-term conditions (LTCs).

Design: Cross-sectional study

Setting: Emergency admissions between 2009 and 2011 recorded in Hospital Episode Statistics Admitted Patient Care data in England.

Participants: 763,199 CYP aged 10-24 years with and without underlying LTC (LTCs were defined using ICD-10 codes recorded in the past five years).

Primary and secondary outcome measures: We calculated emergency admission rates before (10-15 years), and after transition (19-24 years) stratified by gender, LTC, and primary diagnosis. We used negative binomial regression to estimate adjusted incidence rate ratios.

Results: We included 1,109,978 emergency admissions, of which 63.2% were in children with LTCs. The emergency admission rate increased across the age of transition for all CYP, more so for those with LTCs (IRR_{LTC}: 1.55, 99% CI: 1.47-1.63), compared to those without (IRR_{noLTC}: 1.21, 99% CI: 1.18-1.23).

Rates increased most rapidly for CYP with mental health problems, MEDReG (metabolic/endocrine/digestive/renal/genitourinary) disorders, and multiple LTCs (both genders), and respiratory disorders (females only). Small or no increased rates were found for CYP without LTCs and for those with cancer or cardiovascular disease. Increases in length of stay were driven by long admissions (10+ days) for a minority (1%) of CYP with mental health problems and potentially psychosomatic symptoms. Non-specific symptoms related to abdominal pain (girls only), gastrointestinal and respiratory problems were the most frequent primary diagnoses.

For peer review only - http://bmjopen.bmj.com/site/about/guidelines.xhtml

Conclusions: The increased rates and duration of emergency admissions and predominance of non-specific admission diagnoses during transition in CYP with underlying LTCs, may reflect unmet physical or mental health needs.

Strengths and limitations of this study

- This study included all hospital admissions to the NHS in England over three years, minimising selection bias and allowing for comparison of patients using paediatric and adult services.
- Our analysis used a standardised and validated coding system to identify underlying long-term conditions, based on any diagnostic codes recorded at the current or past admissions in the previous five years.
- As we used a cross-sectional design with admissions as the denominator, we did not account for individuals with multiple admissions (i.e. clustering) and could have overestimated the contribution of certain conditions that are associated with multiple emergency admissions.
- We may have included young people who were first diagnosed with long-term conditions during or after the age of transition.

Background

Transition from paediatric to adult services presents challenges to children and young people (CYP), their families and to service providers.[1] Firstly, the team managing the CYP's condition often shifts from specialist, hospital-based to generic, community-based care by general practitioners (GPs). For instance, for diabetes care in the UK, care for children is coordinated by paediatric endocrinologists in hospital, while diabetes care for adults is led by primary care.[2] Additionally, as the clinicians and thresholds for access to specialist care change with the shift to adult services ongoing healthcare is often disrupted. This can be

For peer review only - http://bmjopen.bmj.com/site/about/guidelines.xhtml

For peer review only

particularly problematic for children with long-term conditions (LTCs), who account for 41% of all emergency admission in children in England,[3] as they often have little contact with their primary care physician prior to transition.[4]

Secondly, transition to adult care occurs during a critical and vulnerable period for children with LTCs. Adult services often expect CYP to assume responsibility for their own care despite the fact that psychological, social and physiologic changes associated with adolescence can disrupt self-management of LTCs.[5,6] Finally, the type of clinical problems experienced by CYP during transition are changing due to the increased survival of young children with complex or multiple LTCs partly due to improved care and support for (very) pre-term infants and for CYP with conditions that would previously have been lethal in early childhood.[7,8]

We have previously reported that rates of emergency and elective admissions increase in young people around the time of transition to adult services.[9] Most studies on care across the transition to adult care have used qualitative methods to evaluate patient or clinician expectations and experience,[10] or have studied outcomes in a relatively small, regionally defined or disease specific population.[11,12] Our primary aim was to use cross-sectional analyses of hospital administrative data for England to compare changes in rates and duration of emergency admission to hospital for CYP with and without underlying LTCs before and after the period of transition from paediatric to adult care. In secondary analyses, we explored changes for LTC groups and determined which primary diagnostic groups accounted for changes in admission rates.

For peer review only

Methods

Data source

We used anonymised patient-level Hospital Episode Statistics (HES) data for admissions to English National Health Service (NHS) hospitals from 1 April 2009 to 31 March 2012.[13] We identified admissions as continuous periods in hospital that could consist of several finished consultant episodes (FCEs – a period of hospital stay under a single consultant).[14] Admissions that occurred within a day of each other or admissions that included a hospital transfer were considered as a single admission.

Population

We included adolescents and young people aged 10 to 24 years in our analysis in order to determine incidence rates before (ages 10-15 years) and after (19-24 years) transition. We defined transition from paediatric to adult healthcare between the ages of 16 to 18 year as in practice, the exact age of transition in the NHS can vary between different NHS services and/or conditions.[15–18]

Our main outcome measure was the incidence rate ratio (IRR) of emergency admissions after compared with before the age of transition. We considered emergency (or unplanned) admission to be a clinically important indicator of ill-health, which might be affected by the quality of health care received from hospital or care in the community during transition. We excluded emergency injury admissions from the primary outcome as these would be strongly affected by personal factors or public health or education interventions (e.g. reaching the legal driving and legal drinking age)[9,19] However, injury admissions due to intentional self-harm (identified by a second diagnosis code indicating self-harm[20]) were included as these could signify an underlying mental health problem. We further excluded admissions related to pregnancy and maternities as we expected these to increase with age unrelated to transition from paediatric to adult services.

For peer review only - http://bmjopen.bmj.com/site/about/guidelines.xhtml

Variable definitions

We identified CYP with and without underlying LTCs using validated *International Classification of Diseases, 10th Revision* (ICD-10) codes[14] recorded in any diagnostic code during the index admission or any hospital admission in the previous five years. We defined nine LTC groups: 1) Mental health, 2) Cancer and blood disorders, 3) Chronic infections, 4) Respiratory disorders, 5) Metabolic, endocrine, digestive, renal, genitourinary disorders (MEDReG), 6) Musculoskeletal and skin disorders, 7) Neurological disorders, 8) Cardiovascular disorders, and 9) Multiple LTCs (for children with LTCs from more than one group).

Other characteristics included as potential confounders were ethnicity, quintile of deprivation and whether the CYP was referred by a GP or Emergency Department. Deprivation was based on postcode of residence at admission and measured using the Index of Multiple Deprivation 2004 (IMD).[21] Analyses were stratified by sex.

We used the primary diagnosis at admission to define the main reason for admission grouped as: LTCs (only as primary diagnosis), infection, signs and symptoms, and other (see Appendix B). We calculated length of stay by subtracting the discharge date from the admission date and classified admissions as day cases if admission and discharge occurred on the same date.

Statistical analyses

We calculated incidence rates for the years 2009-2011 per 1,000 person-years by sex, using the number of admissions for each calendar year and age group divided by the mid-year population estimates as the denominator population from the Office of National Statistics[22].The primary analysis determined the change in the incidence of emergency

For peer review only - http://bmjopen.bmj.com/site/about/guidelines.xhtml

inpatient admissions measured by the incidence rate ratio (IRR) for the average incidence after transition (19-24 years) divided by the average incidence before transition (10-15 years).

We determined whether the IRR varied significantly according to whether a child had an underlying LTC recorded in the past five years, as well as by primary diagnosis at admission. To correct these estimates for confounding, we used a negative binomial regression model (to correct for overdispersion) with admission counts as the outcome variable, age, underlying LTC, sex, deprivation quintile and ethnicity as explanatory variables and population size as the offset. We used linear combination of regression coefficients to describe the IRRs for subgroups. To determine the effect of long-term conditions on the increase in admission rates across transition we calculated the population-attributable fraction (PAF).[23]

We conducted sensitivity analyses to assess whether results changed when including admissions related to injury and maternity. In addition, we assessed the effect of changing the criterion for underlying LTCs from being recorded in the previous 5 years to 3 or 1 years.

All analyses were stratified by sex and performed in Stata SE version 13.0 (Statacorp, College Station, Texas, US). Figures were produced using Microsoft Excel.

Results

Overall

We analysed 1,109,978 emergency admissions in 763,199 CYP aged 10-24 years. 680,322 (57.3%) admissions occurred in females. Overall, 701,834 (63.2%) admissions occurred in CYP with a record of a LTC in the last 5 years (eTable A1 in Appendix A). This proportion increased from 57.2% in 10-15 year olds to 66.6% in 19-24 year olds.

The increase in the total rate of emergency admissions (excluding elective admissions and emergency admissions for injury and pregnancy-related problems, eFigure A1 in Appendix

For peer review only - http://bmjopen.bmj.com/site/about/guidelines.xhtml

A) was higher in females than in males: incidence rate ratios comparing average rates after with before transition were 1.64 (99% CI: 1.63-1.64) for females and 1.17 (99% CI: 1.16-1.18) for males. The increase in rates of emergency admission in both sexes occurred predominantly in CYP with underlying LTCs (Figure 1). In the fully adjusted model (Table 1), transition was associated with a modest increase in emergency admission rates for children without LTCs (IRR: 1.21, 99% CI: 1.18-1.23). However, for CYP with LTCs, the effect of transition was stronger (IRR: 1.55, 99% CI: 1.47-1.63).

If CYP with LTCs had the same increase in rates of emergency admission across transition as CYP without LTCs the overall increase in admissions would decrease by 26.3% (population attributable factor (PAF), 99% CI: 25.9% – 26.7%) among females and 25.7% (99% CI: 25.2%– 26.1%) among males.

Inequalities increased across transition (Figure 2). Emergency admission rates varied from 2.2 to 3.6 per 1,000py from most affluent to most deprived quintiles when children were aged 10 years, but this gap increased to 3.8 to 7.0 for 24 year olds.

Admission rates by specific underlying long term condition

The steepest increase in emergency admission rates occurred in CYP who had records indicating underlying mental health problems (Figure 3 eTable A2 in Appendix A). Mental health problems were recorded in 31.2% of emergency admissions in 16-18 year olds, predominantly disorders related to alcohol/drug use, self-harm, depression, eating disorders and schizophrenia. CYP with records indicating MEDReG disorders or multiple LTCs also experienced large increases in admission rates across transition. Emergency admissions for cancer and blood disorders and cardiovascular conditions remained stable or decreased across transition.

For peer review only - http://bmjopen.bmj.com/site/about/guidelines.xhtml

Specific underlying LTCs of asthma, diabetes, and epilepsy increased only marginally, though more so in females (Figures 3 and 4). As expected, larger increases were seen for inflammatory bowel disease (IBD), as this condition often emerges during adolescence. Admissions of CYP with these underlying LTCs (asthma, diabetes, epilepsy or IBD) contributed to a minority of admissions before (14.8%) and after (11.3%) transition. Emergency admissions for these four conditions accounted for 14.1% (PAF 99% CI: 13.8%-14.6%) of the increase across transition, just over half of the total effect (26.0%).

Primary diagnosis on admissions

Although admissions increased in CYP with underling LTCs, the primary reason for admission was often not the LTC itself. We found only modest increases in rates of emergency admission during transition with primary diagnoses recorded for specific LTCs. (Figure 3, eTable A3 in Appendix A). Admissions with a primary diagnosis of asthma or epilepsy remained relatively stable across transition (Figure 4) while IBD increased.

As primary diagnoses for LTCs did not explain the increase in emergency admission rates (eTable A4 in Appendix A), we explored other reasons for admissions. Emergency admissions with primary diagnoses for "signs and symptoms" saw strong increases over the transition. Admissions for abdominal pain, circulatory and respiratory symptoms, and general symptoms all increased substantially. Abdominal pain in particular was the primary reason for emergency admission in 19.7% (for females) and 9.8% (for males) at ages 16-18 years. Overall, emergency admission rates for symptoms increased for both females (IRR: 1.83 (99% CI: 1.81-1.84) and males (IRR: 1.21 (99% CI: 1.20-1.23), eTable A3 in Appendix A).

Emergency admissions for infections also increased (IRR: 1.72 for females, 1.29 for males, eTable A3 in Appendix A). There were also increases in 'any other' diagnoses for emergency

For peer review only - http://bmjopen.bmj.com/site/about/guidelines.xhtml
For peer review only

admissions (where the primary diagnosis was not a LTC, infection nor symptom), although admission rates for individual reasons were low (eTable A3 in Appendix A).

Duration of admission

The length of stay for emergency admissions increased with age from an average of 2.2 days at age 10 years to 4.5 days at age 24 years for females, and from 1.9 to 9.2 for males (Figure 5). The median length of stay was 1 day for the entire age range, for both males and females. While day cases were the most common type of admission at age 10 years (38% for females and 41% for males), there was a drop in the proportion of day cases around the age of transition (age 16-18 years) to 27% for females and 28% for males.

A small proportion of females and males had very long admissions (10 days or longer, n=84,812, 7.1% of total emergency admissions), which increased from 5% before to 7% after transition for females and 4% to 11% for males. A large proportion (18,812, 22.3%) of these long-stay admissions were related to mental health problems such as schizophrenia and personality disorders (eTable A5 in Appendix A), and a minority (4,358, 5.2%) had an unspecified primary diagnosis (ICD-10 code R69.X).

Young adults were more likely to be admitted via the emergency department rather than via their primary care physician or other methods: the proportion of emergency admission via ED increased for females from 51% before transition to 69% after transition and form 53% to 71% for males.

Sensitivity analyses

The results of the sensitivity analyses (Appendix C) show that by excluding injury and maternity/pregnancy related admissions we have underestimated the overall increase in emergency admission rates for females (26%) and males (6%). However, the higher rate of

For peer review only - http://bmjopen.bmj.com/site/about/guidelines.xhtml

admission in females was entirely due to maternity-related emergency admissions which increased from 0.4 to 22.2 per 1,000 person-years.

IRRs for emergency admissions before and after transition for CYP affected by underlying LTCs decreased slightly (from 1.88, 99% CI: 1.87-1.89 to 1.83, 99% CI: 1.82-1.84) when we changed our definition from LTCs recorded in the previous 5 years, to 3 or 1 year.

For peer review only

Discussion

Emergency admission rates increased at the age when young people transition from paediatric to adult healthcare. The increase in emergency admission rates after the age of transition was significantly higher for those with underlying LTCs, compared to CYP without LTCs. Although emergency admissions increased in CYP with underlying LTCs (most steeply for mental health problems, present in 31% of included emergency admissions), the primary diagnosis on admission was often for other problems, most commonly signs and symptoms, and infections. Increases were more marked for females than for males, and for the most deprived CYP.

Average length of stay also increased after transition, particularly for males. A substantial minority of the emergency admissions with long lengths of stay (10+ days) were for mental health conditions: schizophrenia and psychoses for males, and eating and personality disorders for females.

Context

Our results are similar to findings from a study in Ontario, Canada, that found an increase in rates of emergency admissions for young people with diabetes during the transition from paediatric to adult services[12]. The rates for diabetes mellitus-related admissions were lower in our population (1-3 per 1,000 vs. 7-10 per 100 patient-years in Ontario) possibly due to

For peer review only - http://bmjopen.bmj.com/site/about/guidelines.xhtml

differences in the population and in how conditions are coded. However, we found similar trends with regards to deprivation and female gender.

We found that while emergency admission rates for CYP with LTC increased across transition for most sub-groups, rates remained stable for CYP with cardiovascular disorders and cancer or blood disorders. This finding may reflect the intensity of paediatric and adult specialist care for these conditions and a reduced likelihood of emergency admission because other types of hospital care (e.g. clinic review or planned admission) can be readily accessed. Alternatively, transition from paediatric services may be deferred.[24,25] For example, paediatric services for cancer can be commissioned up to age 24 years. A further explanation could be improved transition, e.g. congenital heart disease services have reported using transition nurses to provide continuity between paediatric and adult services [18,25].

Emergency admission rates were more likely to increase in children from more deprived backgrounds, although the effect size was small. We found that after transition emergency admission rates stabilized, both overall and for many LTC groups by age 24 years. Also taking the drop in the proportion of day cases and increase in length of stay into account, this could be explained by a higher admission threshold for adults and potentially unmet health needs as a result.

Strengths and limitations

By analysing all NHS admissions in England, we avoided bias due to different referral pathways for CYP as they transition to adult services. Our analysis of underlying LTCs, based on any diagnostic codes recorded at the current or past admissions in the previous five years, showed that CYP with LTCs often present with primary diagnoses related to symptoms or conditions other than their LTC. Previous studies have focused on well-recognized LTCs such as asthma, diabetes, epilepsy and IBD[26] but in our analyses, these conditions

For peer review only

For peer review only - http://bmjopen.bmj.com/site/about/guidelines.xhtml

accounted for only half of the increase in admissions, and a minority of primary diagnoses. Our finding of relative stability in admissions for cancer and cardiovascular conditions suggests that care over the transition differs between conditions.

The cross-sectional study design of our study is a limitation. As we analysed admissions without taking into account that individuals could have multiple admissions (i.e. clustering) we might have overestimated the contribution of certain conditions that are associated with multiple emergency admissions. Additionally, we may have included young people who were first diagnosed with LTCs after the age of transition.

We used a retrospective capture of past admissions to categorize CYP's emergency admissions into underlying LTC or no LTC. Our analyses of the whole population complement longitudinal analyses that follow individual CYP with and without LTCs across the period of transition. However, longitudinal analyses require identification of inception points, such as first diagnosis of the long-term condition. This is challenging using hospitalization data as many CYP with underlying LTCs such as asthma are mostly managed in primary care. Such analyses would be feasible in future studies using linked primary care and hospitalization data for whole populations.

Implications

Emergency admission may be a manifestation of the widely recognized difficulties young people face when they transition to adult health services.[1,18,27] Emergency admissions are more common and longer after transition, resulting in higher healthcare costs. Our findings of disparities according to presence of underlying LTC and deprivation suggest potential to reduce emergency admissions across transition by focusing on young people with underlying LTCs. This will require system-level strategies and targeted investments.[28]

For peer review only - http://bmjopen.bmj.com/site/about/guidelines.xhtml

The high rates of emergency admissions for symptoms among CYP with LTCs may reflect a common preoccupation among adolescents with 'normal' physical symptoms, which generates more clinical concern and need for investigation in CYP with LTCs.[29,30] Adult care often involves less routine review or anticipatory management of problems and has been reported to be more focused on disease management rather than the CYP's broader health and psychosocial needs.[18] Alternatively, symptoms may reflect undiagnosed complications or morbidity or maybe a manifestation of mental health distress, which might not be investigated in the context of an emergency hospital admission.

A potential unmet need for mental health care is borne out by the steep increase in emergency admission with underlying mental health problems across transition. Previous research has claimed that UK mental health services around transition are not fit for purpose[31] and has called for prioritization of initiatives and research to improve transition care.

Primary care is the first line provider of mental health care for adolescents and the main provider for adults in England and elsewhere. However, rates of GP consultations decline steeply during adolescence, particularly among males[32], also reflected in our study by the decreasing proportion of admissions in older age groups that were referred by the GP. To improve provision of care during transition, services in primary and community care need to be made more attractive and accessible to adolescents to enable improved (mental) health care and thereby reduce the risk of admissions.[33–35] However, although there have been calls to improve primary care involvement in transition care, there is little evidence on the most effective ways to do this.[36]

Hospital specialties in the UK are also making changes to transitional care. Several practice guidelines focus on early planning and communication, and having a single named practitioner to coordinate transition care and support.[4,18,27,37] Our findings suggest that

For peer review only - http://bmjopen.bmj.com/site/about/guidelines.xhtml

future healthcare planning needs to shift from management of transition for specific LTCs to transitional care for the whole person that meets their holistic care needs, particularly mental health, thereby averting serious acute problems that require admission[38].

Abbreviations:

CI – Confidence Interval;
CYP – Children and Young People;
DM – Diabetes Mellitus;
ED – Emergency Department;
FCE – Finished Consultant Episode;
GP – General Practitioner;
HES – Hospital Episode Statistics;
ICD-10 – International Statistical Classification of Diseases and Related Health Problems 10th Revision;
IMD – Index of Multiple Deprivation;
LTCs – Long Term Conditions;
MEDReG disorders – Metabolic, Endocrine, Digestive, Renal or Genitourinary disorders;
NHS – (English) National Health Service;
py – person-year;
RR – Rate Ratio

For peer review only

Funding statement

Dr Wijlaars is funded by the Department of Health Policy Research Programme through funding to the Policy Research Unit in the Health of Children, Young People and Families (grant reference number 109/0001). Dr Hardelid is funded by a National Institute for Health Research postdoctoral fellowship (number PDF-2013-06-004). This article represents independent research funded by the National Institute for Health Research (NIHR) and the Department of Health. The views expressed in this publication are those of the author(s) and not necessarily those of the NHS, the National Institute for Health Research or the Department of Health.

Prof Guttmann is funded by a Canadian Institutes for Health Research Applied Chair Award in Reproductive and Child Health Services Research.

We also acknowledge the support from the Farr Institute of Health Informatics Research (MRC Grant Nos: London MR/ K006584/1).

Competing interests

The authors declare that they have no competing interests.

Authors contributions

Linda Wijlaars: Dr Wijlaars conceptualized and designed the study, carried out the analyses, drafted the initial manuscript, and approved the final manuscript as submitted.

Pia Hardelid: Dr Hardelid helped design the study, reviewed and revised the manuscript, and approved the final manuscript as submitted.

Astrid Guttmann: Prof Guttmann helped conceptualize the study, critically reviewed the manuscript, and approved the final manuscript as submitted.

Ruth Gilbert: Prof Gilbert helped conceptualize and design the study, reviewed and revised the manuscript, and approved the final manuscript as submitted.

All authors approved the final manuscript as submitted and agree to be accountable for all aspects of the work.

Availability of data and material

Use of Hospital Episodes Statistics data was approved by the Health and Social Care Information Centre for the purpose of this study (DARS-NIC-393510-D6H1D-v1.11).

Source data can be accessed by researchers applying to the Health and Social Care Information Centre for England. Copyright © 2017, Re-used with the permission of the Health and Social Care Information Centre. All rights reserved.

Data sharing statement

Technical appendix with details on code list used to identify primary diagnoses group is available in appendix B. Statistical code is available upon request from authors.

For peer review only - http://bmjopen.bmj.com/site/about/guidelines.xhtml

Acknowledgements

We would like to thank Jenny Woodman and David Cottrell for their input into the writing and amending of this manuscript, as well as members of the Policy Research Unit in the Health of Children, Young People and Families: Catherine Law, Russell Viner, Miranda Wolpert, Amanda Edwards, Steve Morris, Helen Roberts, Terence Stephenson, and Cathy Street.

References

1 Crowley R, Wolfe I, Lock K, *et al.* Improving the transition between paediatric and adult healthcare: a systematic review. *Arch Dis Child* 2011;:archdischild202473. doi:10.1136/adc.2010.202473

2 NICE. NG18: Diabetes (type 1 and type 2) in children and young people: diagnosis and management. 2015.http://www.nice.org.uk/guidance/ng18 (accessed 16 Nov 2015).

3 Wijlaars LP, Hardelid P, Woodman J, *et al.* Contribution of recurrent admissions in children and young people to emergency hospital admissions: retrospective cohort analysis of hospital episode statistics. *Arch Dis Child* 2015;:archdischild-2014-307771. doi:10.1136/archdischild-2014-307771

4 Contact a Family. Putting families with disabled children at the heart of the NHS reforms in England (2011). 2012.

5 Suris J-C, Michaud P-A, Viner R. The adolescent with a chronic condition. Part I: developmental issues. *Arch Dis Child* 2004;**89**:938–42. doi:10.1136/adc.2003.045369

6 Sawyer SM, Drew S, Yeo MS, *et al.* Adolescents with a chronic condition: challenges living, challenges treating. *The Lancet* 2007;**369**:1481–9. doi:10.1016/S0140-6736(07)60370-5

7 Cohen E, Kuo DZ, Agrawal R, *et al.* Children with medical complexity: an emerging population for clinical and research initiatives. *Pediatrics* 2011;**127**:529–38. doi:10.1542/peds.2010-0910

8 Larroque B, Bréart G, Kaminski M, *et al.* Survival of very preterm infants: Epipage, a population based cohort study. *Arch Dis Child - Fetal Neonatal Ed* 2004;**89**:F139–44. doi:10.1136/adc.2002.020396

9 Wijlaars L, Hardelid P, Woodman J, *et al.* Contribution of recurrent admissions in children and young people to emergency hospital admissions: retrospective cohort analysis of hospital episode statistics. *Arch Dis Child* in press.

10 Scal P, Ireland M. Addressing Transition to Adult Health Care for Adolescents With Special Health Care Needs. *Pediatrics* 2005;**115**:1607–12. doi:10.1542/peds.2004-0458

11 Reid GJ, Irvine MJ, McCrindle BW, *et al.* Prevalence and Correlates of Successful Transfer From Pediatric to Adult Health Care Among a Cohort of Young Adults With Complex Congenital Heart Defects. *Pediatrics* 2004;**113**:e197–205.

For peer review only - http://bmjopen.bmj.com/site/about/guidelines.xhtml

12 Nakhla M, Daneman D, To T, *et al.* Transition to Adult Care for Youths With Diabetes Mellitus: Findings From a Universal Health Care System. *Pediatrics* 2009;**124**:e1134–41. doi:10.1542/peds.2009-0041

13 Health and Social Care Information Centre 1 Trevelyan Square. Hospital Episode Statistics. 2012.http://www.hscic.gov.uk/hes (accessed 28 Aug 2014).

14 Hardelid P, Dattani N, Davey J, *et al.* Overview of child deaths in the four UK countries. RCPCH 2013. bit.ly/hardelidreport

15 Kennedy I. Getting it right for children and young people: overcoming cultural barriers in the NHS so as to meet their needs. Department of Health 2010. https://www.gov.uk/government/publications/getting-it-right-for-children-and-young-people-overcoming-cultural-barriers-in-the-nhs-so-as-to-meet-their-needs

16 Barnes D, Wistow R, Dean R, *et al.* National child and adolescent mental health service mapping exercise 2005. Durham University, School ofApplied Social Sciences 2006. http://dro.dur.ac.uk/4333/1/4333.pdf?DDD34+dss4ae+dul0jk

17 Davies SC. Prevention pays - our children deserve beter. 2013. https://www.gov.uk/government/news/chief-medical-officer-prevention-pays-our-children-deserve-better

18 Care Quality Commission. From the pond into the sea: Children's transition to adult health services. 2014. https://admin.cqc.org.uk/sites/default/files/CQC_Transition%20Report.pdf

19 Gonzalez-Izquierdo A, Cortina-Borja M, Woodman J, *et al.* Maltreatment or violence-related injury in children and adolescents admitted to the NHS: comparison of trends in England and Scotland between 2005 and 2011. *BMJ Open* 2014;**4**:e004474. doi:10.1136/bmjopen-2013-004474

20 Clinical Classifications Service. Chapter XX External Causes of Morbidity and Mortality (V01-Y98). In: *National Clinical Coding Standards ICD-10 5th Edition (2016)*. Leeds: : Health & Social Care Information Centre 2016.

21 Government D for C and L. The English Indices of Deprivation 2004 (revised). 2007.http://webarchive.nationalarchives.gov.uk/20120919132719/http://www.communities.gov.uk/publications/communities/englishindices (accessed 17 Sep 2015).

22 Population Estimates for UK, England and Wales, Scotland and Northern Ireland. Off. Natl. Stat. 2011.http://www.ons.gov.uk/ons/rel/pop-estimate/population-estimates-for-uk--england-and-wales--scotland-and-northern-ireland/population-estimates-timeseries-1971-to-current-year/index.html (accessed 27 Mar 2014).

23 Greenland S, Drescher K. Maximum Likelihood Estimation of the Attributable Fraction from Logistic Models. *Biometrics* 1993;**49**:865–72. doi:10.2307/2532206

24 Cystic Fibrosis Trust. Transition from paediatric to adult care. A guide for commissioners, hospital and clincal team. Bromley: 2013. https://www.cysticfibrosis.org.uk/~/media/documents/life-with-cf/publications/factsheet-transition-to-adult-care-for-commissioners.ashx?la=en

For peer review only - http://bmjopen.bmj.com/site/about/guidelines.xhtml

25 Smith S, Case L, Waterhouse K, *et al.* A blueprint of care for teenagers and young adults with cancer. Teenage Cancer Trust 2012. https://www.teenagecancertrust.org/sites/default/files/Blueprint-of-Care.pdf

26 Cohen E, Gandhi S, Toulany A, *et al.* Health Care Use During Transfer to Adult Care Among Youth With Chronic Conditions. *Pediatrics* 2016;**137**:e20152734. doi:10.1542/peds.2015-2734

27 NICE. NG 43: Transition from children's to adults' services. 2016. https://www.nice.org.uk/guidance/indevelopment/gid-scwave0714 (accessed 16 Nov 2015).

28 Hepburn CM, Cohen E, Bhawra J, *et al.* Health system strategies supporting transition to adult care. *Arch Dis Child* 2015;**100**:559–64. doi:10.1136/archdischild-2014-307320

29 Garber J, Walker LS, Zeman J. Somatization symptoms in a community sample of children and adolescents: Further validation of the Children's Somatization Inventory. *Psychol Assess J Consult Clin Psychol* 1991;**3**:588–95. doi:10.1037/1040-3590.3.4.588

30 Eminson M, Benjamin S, Shortall A, *et al.* Physical Symptoms and Illness Attitudes in Adolescents: An Epidemiological Study. *J Child Psychol Psychiatry* 1996;**37**:519–28. doi:10.1111/j.1469-7610.1996.tb01438.x

31 Memarzia J, Clair MCS, Owens M, *et al.* Adolescents leaving mental health or social care services: predictors of mental health and psychosocial outcomes one year later. *BMC Health Serv Res* 2015;**15**:185. doi:10.1186/s12913-015-0853-9

32 Wang Y, Hunt K, Nazareth I, *et al.* Do men consult less than women? An analysis of routinely collected UK general practice data. *BMJ Open* 2013;**3**:e003320. doi:10.1136/bmjopen-2013-003320

33 Zimmer-Gembeck MJ, Alexander T, Nystrom RJ. Adolescents report their need for and use of health care services. *J Adolesc Health* 1997;**21**:388–99. doi:10.1016/S1054-139X(97)00167-5

34 Klein JD, McNulty M, Flatau CN. Adolescents' access to care: Teenagers' self-reported use of services and perceived access to confidential care. *Arch Pediatr Adolesc Med* 1998;**152**:676–82.

35 Booth ML, Bernard D, Quine S, *et al.* Access to health care among Australian adolescents young people's perspectives and their sociodemographic distribution. *J Adolesc Health* 2004;**34**:97–103. doi:10.1016/j.jadohealth.2003.06.011

36 Bhawra J, Toulany A, Cohen E, *et al.* Primary care interventions to improve transition of youth with chronic health conditions from paediatric to adult healthcare: a systematic review. *BMJ Open* 2016;**6**:e011871. doi:10.1136/bmjopen-2016-011871

37 Rosen DS, Blum RW, Britto M, *et al.* Transition to adult health care for adolescents and young adults with chronic conditions: position paper of the Society for Adolescent Medicine. *J Adolesc Health* 2003;**33**:309–311.

For peer review only - http://bmjopen.bmj.com/site/about/guidelines.xhtml

38 Davis AM, Brown RF, Taylor JL, *et al.* Transition Care for Children With Special Health Care Needs. *Pediatrics* 2014;**134**:900–8. doi:10.1542/peds.2014-1909

For peer review only

For peer review only - http://bmjopen.bmj.com/site/about/guidelines.xhtml

Tables

Table 1: Relative admission rates for CYP with and without LTCs and before (10-15 years) and after (19-24 years) transition

	**No LTC**	**LTC**	**No LTC**	**LTC**
**Covariate**	crude IRR (99% CI)		adjusted IRR (99% CI)	
Transition	1.18 (1.17-1.18)	1.44 (1.41 – 1.47)	1.21 (1.18 – 1.23)	1.55 (1.47 – 1.63)
Sex (reference = males)	1.07 (1.06 – 1.07)	0.92 (0.90 – 0.94)	1.05 (1.05 – 1.06)	0.98 (0.96 – 1.00)
Age	1.02 (1.01 – 1.02)	1.03 (1.03 – 1.04)	1.00 (0.99 – 1.00)	0.98 (0.98 – 0.99)
IMD '04				
1 - Most deprived	1.10 (1.09 – 1.11)	1.41 (1.36 – 1.46)	1.07 (1.05 – 1.08)	1.17 (1.13 – 1.20)
2	1.07 (1.06 – 1.09)	1.28 (1.23 – 1.32)	1.04 (1.03 – 1.06)	1.08 (1.05 – 1.12)
3	1.07 (1.06 – 1.10)	1.05 (1.01 – 1.09)	1.07 (1.05 – 1.08)	1.02 (0.99 – 1.05)
4	1.02 (1.01 – 1.04)	1.04 (1.00 – 1.07)	1.02 (1.00 – 1.03)	1.02 (0.98 – 1.05)
5 – Least deprived	Reference	Reference	Reference	Reference
Missing	0.88 (0.86 – 0.91)	1.11 (1.01 - 1.22)	0.87 (0.85 – 0.89)	1.01 (0.92 – 1.10)
Ethnicity				
White	Reference	Reference	Reference	Reference
Black	1.21 (1.18 – 1.24)	2.67 (2.56 – 2.79)	1.19 (1.16 – 1.22)	2.49 (2.39 – 2.60)
Asian	1.03 (1.01 – 1.04)	1.07 (1.03 – 1.12)	1.03 (1.01 – 1.04)	1.13 (1.08 – 1.19)
Mixed	0.94 (0.92 – 0.95)	0.93 (0.89 – 0.97)	0.93 (0.92 – 0.95)	0.93 (0.89 – 0.97)
Unknown	0.75 (0.74 – 0.77)	0.43 (0.39 – 0.47)	0.75 (0.74 – 0.77)	0.43 (0.40 – 0.47)
Missing	0.82 (0.80 – 0.84)	0.52 (0.49 – 0.54)	0.82 (0.81 – 0.84)	0.54 (0.51 – 0.57)

IRR = incidence rate ratio, the IRR compares the emergency admission rate before (age 10-15 years) transition with the rate after (age 19-24 years) transition.
Age is included in the model as a continuous variable IRR refers to increase per year of age.
Models are corrected for variables shown in table.

Figures

Figure 1: Emergency and elective admission rates by age and sex
LTCs = long-term conditions as measured in the index admission and admissions in the previous 5 years.
Grey area indicates age range for transition

For peer review only - http://bmjopen.bmj.com/site/about/guidelines.xhtml

Figure 2: Emergency admissions by Index of Multiple Deprivation (IMD04) quintile
Grey area indicates age range for transition

Figure 3: Incidence rate ratios for emergency admissions before (10-15 years) and after (19-24 years) transition by underlying long-term condition (LTC) groups and sex.
Marker size represents proportion of admissions during transition (age 16-18 years) with a record of LTCs (displayed on the right). Diamonds indicate group/overall estimates. IRR = incidence rate ratio, MEDReG disorders: Metabolic, Endocrine, Digestive, Renal or Genitourinary disorders

Figure 4: Incidence rate ratios for emergency admissions before (10-15 years) and after (19-24 years) transition by primary diagnosis and sex.
Marker size represents proportion of admissions at age 16-18 years with each primary diagnosis. The 15 primary diagnoses shown represent 76.9% and 77.7% of all primary diagnoses for females and males, respectively. LTCs are highlighted in bold. IRR = incidence rate ratio

*Diseases of male genital organs (ICD-10 codes N40-N51) include torsion of testis, and inflammatory diseases of the male genital organs such as orchitis and epididymitis.

Figure 5: Average length of stay per admission by patient age
Grey area indicates age range for transition

For peer review only

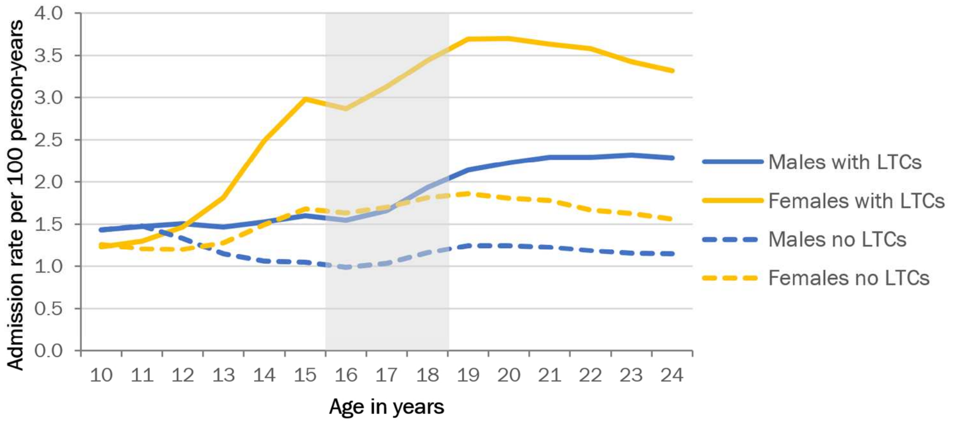

Emergency and elective admission rates by age and sex
LTCs = long-term conditions as measured in the index admission and admissions in the previous 5 years.
Grey area indicates age range for transition

149x65mm (300 x 300 DPI)

For peer review only

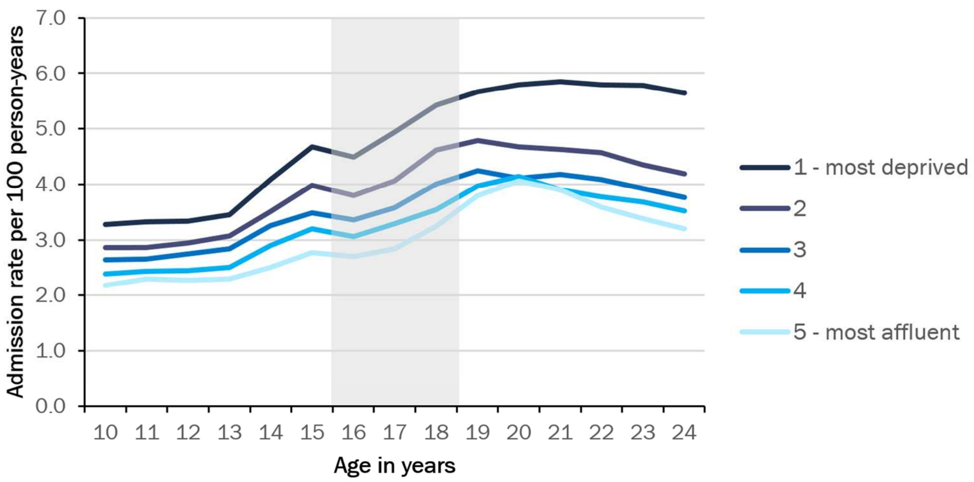

Emergency admissions by Index of Multiple Deprivation (IMD04) quintile
Grey area indicates age range for transition

132x64mm (300 x 300 DPI)

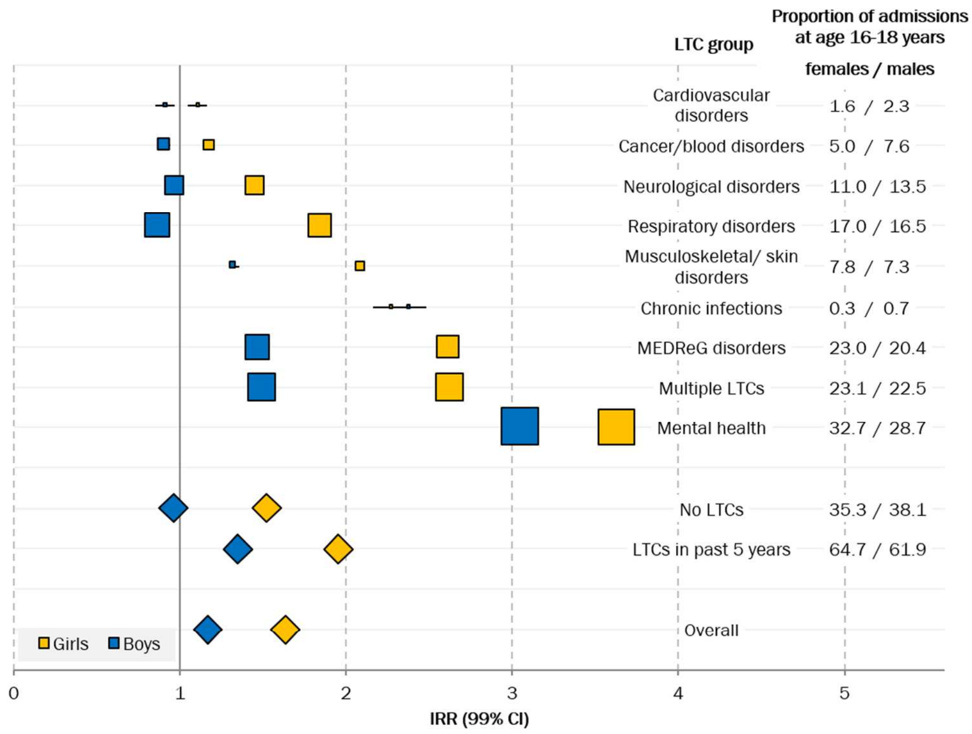

Incidence rate ratios for emergency admissions before (10-15 years) and after (19-24 years) transition by underlying long-term condition (LTC) groups and sex.
Marker size represents proportion of admissions during transition (age 16-18 years) with a record of LTCs (displayed on the right). Diamonds indicate group/overall estimates. IRR = incidence rate ratio, MEDReG disorders: Metabolic, Endocrine, Digestive, Renal or Genitourinary disorders

96x71mm (300 x 300 DPI)

only

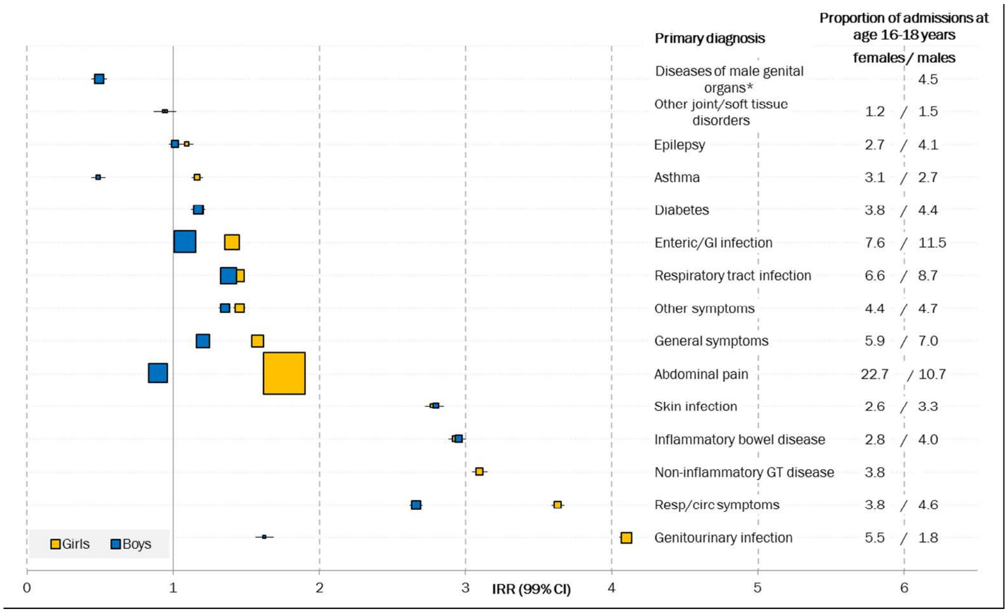

Incidence rate ratios for emergency admissions before (10-15 years) and after (19-24 years) transition by primary diagnosis and sex.
Marker size represents proportion of admissions at age 16-18 years with each primary diagnosis. The 15 primary diagnoses shown represent 76.9% and 77.7% of all primary diagnoses for females and males, respectively. LTCs are highlighted in bold. IRR = incidence rate ratio
*Diseases of male genital organs (ICD-10 codes N40-N51) include torsion of testis, and inflammatory diseases of the male genital organs such as orchitis and epididymitis.

122x73mm (300 x 300 DPI)

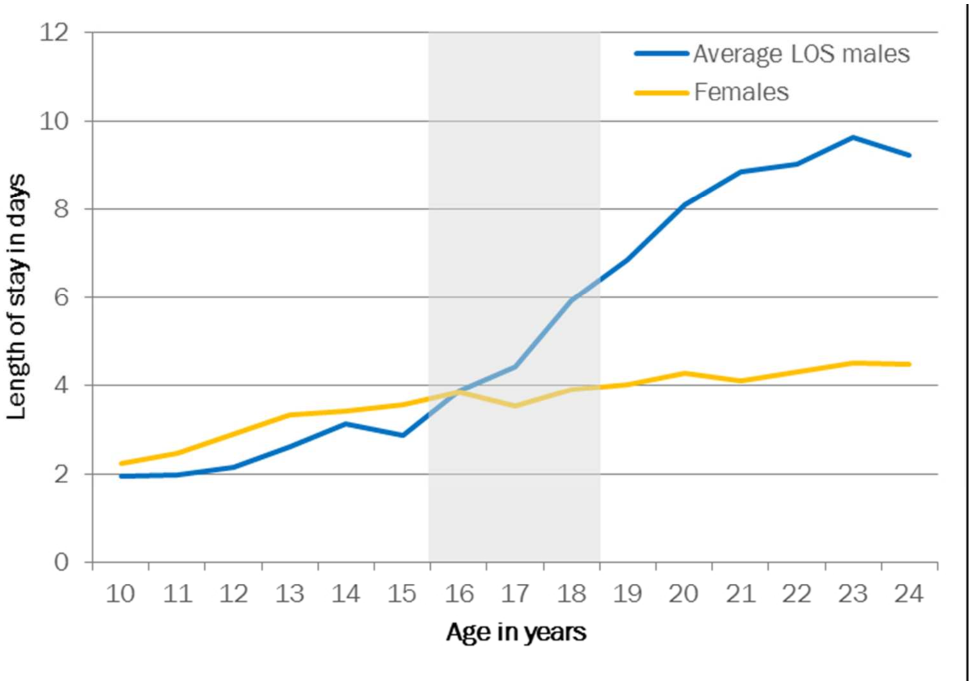

Average length of stay per admission by patient age
Grey area indicates age range for transition

66x44mm (300 x 300 DPI)

Appendix A: additional tables and figures

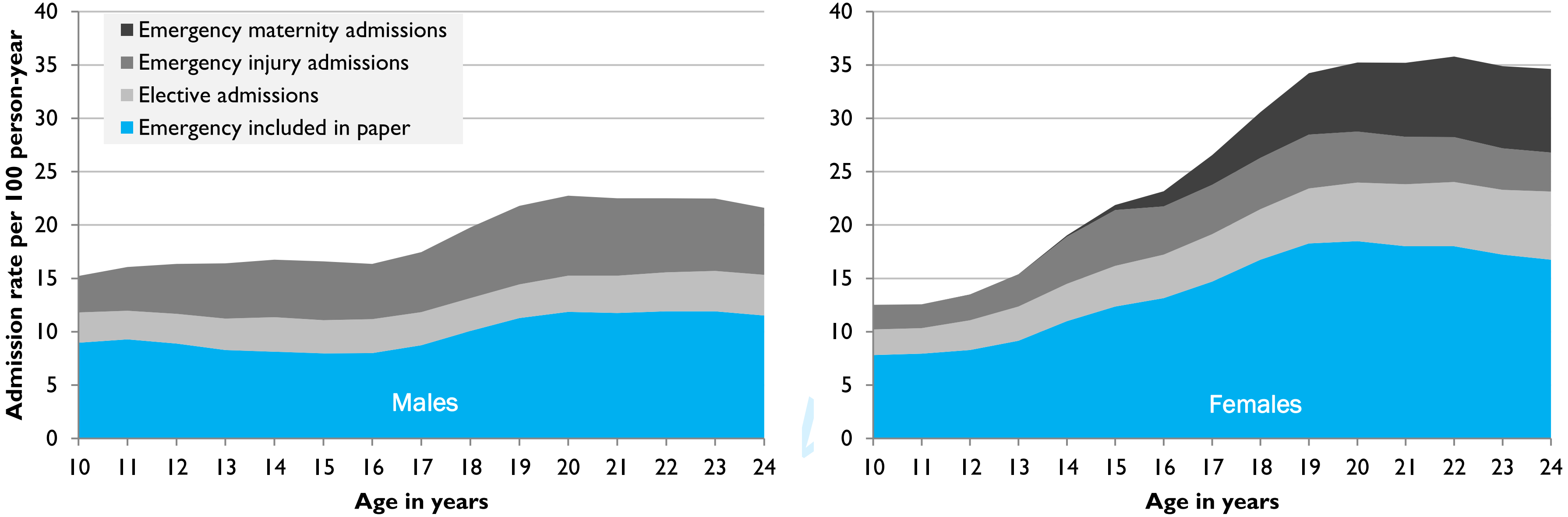

Figure A1: Emergency admission included in context: emergency admissions included in the primary analyses are shown in blue, while excluded admissions (elective admissions, emergency injury and maternity admissions) are shown in grey.

**Table A1:** Baseline characteristics of included admissions			
	**Overall**	**No LTC**	**LTC**
**Covariate**	**Number of admissions (%)**		
**Total**	**1,109,978**	**410,144**	**699,834**
Before transition (10-15 years)	327,531 (29.5)	142,206 (34.7)	185,325 (26.5)
During transition (16-18 years)	224,722 (20.3)	81,676 (19.9)	143,046 (20.4)
After transition (19-24 years)	557,725 (50.3)	186,262 (45.4)	371,463 (53.1)
Females	644,994 (58.1)	229,651 (56.0)	415,343 (59.4)
IMD '04			
1 - Most deprived	319,837 (28.8)	108,211 (26.4)	211,626 (30.2)
2	242,428 (21.8)	85,930 (21.0)	156,498 (22.4)
3	198,237 (17.9)	74,583 (18.2)	123,690 (17.7)
4	172,479 (15.5)	68,364 (16.7)	104,115 (14.9)
5 – Least deprived	159,588 (14.4)	65,817 (16.1)	93,771 (13.4)
Missing	17,373 (1.6)	7,239 (1.8)	10,134 (1.5)
Ethnicity			
White	860,552 (77.5)	307,092 (74.9)	553,460 (79.1)
Black or Black British	45,564 (4.1)	14,093 (3.4)	31,471 (4.5)
Asian or Asian British	75,393 (6.8)	30,460 (7.4)	44,933 (6.4)
Mixed	44,863 (4.0)	17,414 (4.3)	27,449 (3.9)
Unknown	20,251 (1.8)	10,118 (2.5)	10,133 (1.5)
Missing	63,355 (5.7)	30,967 (7.6)	32,388 (4.6)

For peer review only

Table A2: Admission rates, incidence rate ratios and trends in rates before, during, and after transition to adult health care for children with and without underlying long-term conditions (LTCs)

	Before transition		After transition		Before	During	After
	Percentage of admissions 10-15 years	Average rate 10-15 years	Average rate 19-24 years		Trend age 10-15 years	Trend age 16-18 years	Trend age 19-25 years
**FEMALES**		Rate per 1,000 person-years		IRR (99% CI)†	Annual change in admission rate per 1,000 person-years		
**Overall***	**100**	**32.5**	**52.7**	**1.62 (1.61 – 1.63)**	**↑4.48**	**↑3.78**	**↓-1.39**
No LTCs	41.8	13.6	17.1	1.26 (1.25 – 1.28)	↗0.87	↗0.92	↘-0.61
History of LTCs†	58.2	18.9	35.5	1.88 (1.87 – 1.89)	↑3.61	↑2.86	↘-0.78
**LTC groups**							
Mental health disorders	20.5	6.7	17.8	2.67 (2.65 – 2.68)	↑2.87	↑1.48	↘-0.63
Substance use	2.9	0.9	4.6	4.94 (4.90 – 4.98)	↗0.51	↗0.77	→-0.09
Cancer/blood disorders	6.8	2.2	2.6	1.20 (1.17 – 1.23)	→0.09	→-0.02	→0.00
Chronic infections	0.4	0.1	0.3	2.34 (2.22 – 2.45)	→0.02	→0.02	→0.01
Respiratory disorders	16.3	5.3	10.3	1.94 (1.92 – 1.96)	↗0.53	↑1.11	↘-0.23
Asthma	11.4	3.7	6.6	1.79 (1.77 – 1.81)	↗0.29	↗0.79	↘-0.20
MEDReG‡ disorders	18.8	6.1	16.2	2.66 (2.64 – 2.67)	↗0.66	↑2.12	↗0.15
Inflammatory bowel disease	3.2	1.0	2.4	2.35 (2.31 – 2.39)	→0.05	↗0.30	→0.04
Diabetes	5.2	1.7	2.0	1.16 (1.13 – 1.20)	↗0.19	↗0.13	↘-0.15
Musculoskeletal/skin disorders	7.7	2.5	5.3	2.11 (2.09 – 2.14)	↗0.33	↗0.40	→0.05
Neurological disorders	13.1	4.3	6.5	1.52 (1.50 – 1.54)	↗0.44	↗0.23	→0.06
Epilepsy	5.2	1.7	1.9	1.13 (1.09 – 1.21)	↗0.11	→-0.02	→-0.04
Cardiovascular disorders	2.1	0.7	0.8	1.16 (1.10 – 1.21)	→0.05	→-0.07	→-0.01
Multiple LTCs	17.8	5.8	15.2	2.62 (2.61 – 2.64)	↑1.00	↑1.66	→-0.05
**MALES**					Annual change in admission rate per 1,000 person-years		
**Overall***	**100**	**27.5**	**34.6**	**1.26 (1.25 – 1.27)**	**↘-0.70**	**↑2.80**	**→0.07**
No LTCs	45.3	12.4	12.0	0.96 (0.95 – 0.98)	↘-0.97	↗0.88	↘-0.23

For peer review only

History of LTCs†	54.7	15.0	22.6	1.50 (1.49 – 1.51)	↗0.27	↑1.91	↗0.30
**LTC groups**							
Mental health disorders	13.4	3.7	12.6	3.43 (3.41 – 3.45)	↗0.61	↑1.75	↗0.30
Substance use	2.0	0.5	4.9	9.01 (8.97 – 9.06)	↗0.29	↗0.98	↗0.22
Cancer/blood disorders	8.8	2.4	2.2	0.92 (0.89 – 0.96)	→0.02	→-0.07	→0.02
Chronic infections	0.5	0.1	0.5	2.44 (2.34 – 2.55)	→0.01	→0.02	→0.05
Respiratory disorders	19.0	5.2	4.9	0.94 (0.92 – 0.96)	↘-0.33	↗0.17	→-0.08
Asthma	13.8	3.8	3.1	0.82 (0.79 – 0.84)	↘-0.36	↗0.14	→-0.09
MEDReG‡ disorders	19.1	5.2	7.9	1.51 (1.49 – 1.53)	↗0.11	↗0.54	↗0.28
Inflammatory bowel disease	4.2	1.1	1.9	1.62 (1.58 – 1.66)	→-0.03	↗0.18	→0.05
Diabetes	4.7	1.3	1.4	1.08 (1.04 – 1.12)	↗0.11	↗0.16	→-0.06
Musculoskeletal/skin disorders	6.8	1.9	2.6	1.38 (1.35 – 1.41)	→0.08	→0.08	→0.10
Neurological disorders	14.5	4.0	4.1	1.04 (1.02 – 1.06)	→0.00	→-0.03	→0.07
Epilepsy	5.9	1.6	1.8	1.14 (1.10 – 1.17)	→-0.04	→0.00	→0.02
Cardiovascular disorders	2.6	0.7	0.7	0.94 (0.88 – 0.99)	→-0.02	→-0.06	→0.02
Multiple LTCs	19.0	5.2	8.5	1.63 (1.61 – 1.64)	↗0.19	↗0.55	↗0.23

*Excluding admissions for injury or maternity; †At least 1 admission with a record of a long-term condition in the previous 5 years (including current admission); ‡Metabolic, endrocrine, digestive, renal or genitourinary disorders

†Rate ratio comparing emergency admission rates before and after transition

Trends:

↑ increasing by ≥1.0 admission per 1,000 person-year (py);

↗ increasing by 0.1-0.99 per 1,000py;

→ stable at -0.1 to 0.1 per 1,000 py;

↘ decreasing by -0.1 to -0.99 per 1,000py;

↓ decreasing by ≤-1.0 per 1,000py

For peer review only

Table A3: Admission rates, incidence rate ratios and trends in rates before, during, and after transition to adult health care by primary diagnosis

	Proportion of admissions 10-15 years	Average rate 10-15 years	Average rate 19-24 years	IRR (99% CI)†	Trend age 10-15 years	Trend age 16-18 years	Trend age 19-25 years
**FEMALES***	Rate per 1,000 person-years				Annual change in admission rate per 1,000 person-years		
**Long-term conditions**							
Any long-term condition	26.4	9.8	19.8	2.02 (2.00 – 2.03)	↑1.04	↑2.54	↘-0.12
Diabetes	3.7	1.2	1.4	1.18 (1.13 – 1.22)	→0.10	↗0.26	↘-0.22
Asthma	4.5	1.4	1.6	1.17 (1.13 – 1.20)	↘-0.11	↗0.14	→-0.02
Inflammatory bowel disease	2.3	0.7	2.1	2.93 (2.88 – 2.98)	→0.03	↗0.37	→0.03
Epilepsy	3.4	1.1	1.2	1.09 (1.05 – 1.14)	→0.07	→0.02	→-0.06
**Infection**							
Any infection	25.4	8.4	14.4	1.72 (1.70 – 1.73)	↗0.26	↑1.66	↘-0.50
Enteric/GI infection	9.4	2.9	4.1	1.40 (1.38 – 1.43)	→0.06	↗0.28	↘-0.11
Respiratory tract infection	7.0	2.2	3.1	1.45 (1.42 – 1.48)	→0.05	↗0.30	↘-0.13
Genitourinary infection	2.5	0.8	3.2	4.10 (4.05 – 4.14)	↗0.11	↗0.58	↘-0.14
**Symptoms**							
Any symptoms	35.8	11.3	20.5	1.83 (1.81 – 1.84)	↑1.31	↑1.95	↘-0.48
Abdominal pain	22.6	7.1	12.5	1.76 (1.75 – 1.78)	↗0.79	↑1.26	↘-0.46
General symptoms	6.9	2.2	3.4	1.58 (1.55 – 1.61)	↗0.31	↗0.12	→-0.04
**Other**							
Any other	12.4	2.6	4.9	1.87 (1.85 – 1.90)	↗0.33	↗0.44	→0.06
Non-inflammatory disease of the female GT	2.3	0.6	1.8	3.10 (3.04 – 3.15)	↗0.22	↗0.20	→-0.04
Other joint/soft tissue disorders	2.4	0.5	0.5	0.95 (0.89 – 1.02)	→-0.01	→0.04	→0.00
**MALES***					Annual change in admission rate per 1,000 person-years		

For peer review only

**Long-term conditions**							
Any long-term condition	25.8	8.9	14.4	1.61 (1.60 – 1.62)	↗0.17	↑1.50	↗0.29
Diabetes	3.3	0.9	1.1	1.17 (1.12 – 1.22)	→0.09	↗0.21	↘-0.13
Asthma	5.5	1.5	0.8	0.49 (0.44 – 0.53)	↘-0.29	→-0.05	→-0.03
Inflammatory bowel disease	2.9	0.7	2.0	2.95 (2.91 – 3.00)	→0.02	↗0.24	→0.01
Epilepsy	4.1	1.2	1.2	1.01 (0.97 – 1.06)	→-0.01	→0.05	→-0.01
**Infection**							
Any infection	28.6	8.5	10.9	1.29 (1.27 – 1.30)	↘-0.47	↑1.08	↘-0.14
Enteric/GI infection	12.7	3.6	3.9	1.08 (1.06 – 1.11)	↘-0.15	↗0.15	→-0.06
Respiratory tract infection	7.0	2.0	2.7	1.38 (1.35 – 1.41)	↘-0.13	↗0.42	↘-0.11
Genitourinary infection	1.7	0.5	0.8	1.63 (1.57 – 1.69)	→-0.03	↗0.11	→-0.01
**Symptoms**							
Any symptoms	29.2	8.3	10.1	1.21 (1.20 – 1.23)	↘-0.52	↗0.97	→0.05
Abdominal pain	15.4	4.4	4.0	0.90 (0.88 – 0.92)	↘-0.54	↗0.28	→-0.02
General symptoms	6.8	1.9	2.3	1.20 (1.17 – 1.24)	→0.02	↗0.19	→-0.01
**Other**							
Any other	16.4	3.4	3.7	1.10 (1.08 – 1.13)	↘-0.21	↗0.26	→0.02
Diseases of male genital organs	6.0	1.2	0.6	0.49 (0.44 – 0.55)	→-0.08	→-0.03	→-0.04
Other joint/soft tissue disorders	2.3	0.5	0.4	0.94 (0.87 – 1.01)	→-0.06	→0.00	→0.02

*Excluding admissions for injury or maternity

†Incidence rate ratio comparing emergency admission rates before and after transition

Trends:

↑ increasing by ≥1.0 admission per 1,000 person-year (py);

↗ increasing by 0.1-0.99 per 1,000py;

→ stable at -0.1 to 0.1 per 1,000 py;

↘ decreasing by -0.1 to -0.99 per 1,000py;

↓ decreasing by ≤-1.0 per 1,000py

For peer review only

Table A4: Most frequent diagnoses for CYP with and without underlying LTCs

Rank	CYP with LTCs			CYP without LTCs		
	**10-15 years**	**16-18 years**	**19-24 years**	**10-15 years**	**16-18 years**	**19-24 years**
	Diagnosis group* (frequency, % of admissions)			Diagnosis group* (frequency, % of admissions)		
1	Asthma (13,209, 7.1%)	Abdominal and pelvic pain (14,643, 10.2%)	Abdominal and pelvic pain (41,809, 11.3%)	Abdominal and pelvic pain (34,348, 24.2%)	Abdominal and pelvic pain (19,231, 23.6%)	Abdominal and pelvic pain (39,952, 21.5%)
2	Abdominal and pelvic pain (13,015, 7.0%)	Poisoning by nonopioid analgesics, antipyretics and antirheumatics (12,835, 9.0%)	Poisoning by nonopioid analgesics, antipyretics and antirheumatics (20,632, 5.6%)	Acute appendicitis (11,699, 8.2%)	Acute appendicitis (6,266, 7.7%)	Pain in throat and chest (10,574, 5.7%)
3	Insulin-dependent diabetes mellitus (10,277, 5.6%)	Insulin-dependent diabetes mellitus (6,694, 4.7%)	Pain in throat and chest (10,737, 2.9%)	Other functional intestinal disorders (4,783, 3.4%)	Acute tonsillitis (4,272, 5.2%)	Acute appendicitis (10,206, 5.5%)
4	Poisoning by nonopioid analgesics, antipyretics and antirheumatics (10,105, 5.5%)	Asthma (4,191, 2.9%)	Other noninfective gastroenteritis and colitis (10,546, 2.8%)	Viral infection of unspecified site (4,615, 3.3%)	Pain in throat and chest (3,105, 3.8%)	Headache (6,984, 3.8%)
5	Epilepsy (6,146, 3.3%)	Mental and behavioral disorders due to use of alcohol (3,796, 2.7%)	Insulin-dependent diabetes mellitus (9,971, 2.7%)	Acute tonsillitis (4,275, 3.0%)	Other disorders of urinary system (3,046, 3.7%)	Other disorders of urinary system (6,029, 3.2%)

*Diagnoses are grouped by 3-character ICD codes (e.g. E84 for cystic fibrosis)
Signs and symptoms are highlighted

For peer review only

Table A5: Most frequent diagnoses for long (10+ days) lengths of stay by age group and gender

Rank	Males: 10-15 years	Males: 16-18 years	Males: 19-24 years	Females: 10-15 years	Females: 16-18 years	Females: 19-24 years
	Diagnosis group* (frequency, *average length of stay*)			Diagnosis group* (frequency, *average length of stay*)		
1	Cystic Fibrosis (414, *8.6*)	Unknown and unspecified causes of morbidity (354, ***53.4***)	Schizophrenia (3,268, ***140.0***)	Cystic fibrosis (612, *8.6*)	Cystic fibrosis (390, *8.8*)	Abdominal and pelvic pain (1,111, *2.0*)
2	Pneumonia, organism unspecified (205, *4.6*)	Pneumothorax (313, *6.0*)	Unknown and unspecified causes of morbidity (2,196, ***97.6***)	Eating disorders (519, ***41.9***)	Abdominal and pelvic pain (386, *1.8*)	Unknown and unspecified causes of morbidity (1,096, ***40.5***)
3	Lymphoid leukaemia (193, *4.9*)	Schizophrenia (268, ***119.7***)	Acute and transient psychotic disorders (773, ***44.3***)	Pneumonia, organism unspecified (221, *4.6*)	Unknown and unspecified causes of morbidity (328, ***53.4***)	Specific personality disorders (917, ***32.5***)
4	Osteomyelitis (175, ***11.0***)	Cystic Fibrosis (254, *8.8*)	Crohn disease (648, *7.4*)	Lymphoid leukaemia (164, *4.9*)	Eating disorders (316, ***49.5***)	Cystic fibrosis (761, ***11.4***)
5	Unspecified acute lower respiratory infection / Acute appendicitis (171, *3.2* / *3.1*)	Ulcerative colitis (240, *8.9*)	Ulcerative colitis (590, *8.7*)	Unspecified acute lower respiratory infection (160, *3.2*)	Crohn disease (211, *7.0*)	Crohn disease (721, *7.4*)

*Diagnoses are grouped by 3-character ICD codes (e.g. E84 for cystic fibrosis)
Average lengths of stay are calculated for all emergency admissions (excluding injury and maternity-related admissions). Average lengths of stay of 10 days or greater are shown in bold.
Mental health conditions are highlighted in blue, unspecified conditions and conditions that could represent psychosomatic symptoms are highlighted in yellow.

Appendix B: Code lists

In order to identify primary diagnoses groups in Table 2 and Figures in Appendix C, we used the following ICD-10 codes:

Table B1: ICD-10 codes used to define condition groups

Primary diagnoses group	ICD-10 codes
Long-term conditions	
Diabetes	E10-E14, G59.0, G63.2, I79.2, M14.2, N08.3, O24.0, O24.1, O24.2, O24.3, Y42.3
Asthma and chronic lower respiratory disease	J41-J47
Epilepsy	F80.3, G40.0, G40.1, G40.2, G40.3, G40.4, G40.6, G40.7, G41, G40.8, G40.9, R56.8, Y46.0, Y46.1, Y46.2, Y46.3, Y46.4, Y46.5, Y46.6
Non-infective enteritis and colitis / inflammatory bowel disease	K50, K51, K52
Cystic fibrosis	E84, P75
Sickle cell disease	D57
Mental health conditions	
Substance use	F10-F19
Schizophrenia, schizotypical and delusional disorders	F20-F29
Personality disorders	F60, F61, F69
Mood disorders	F30-F39
Infections	
Enteric / gastrointestinal infection	A00-A09, I88.0, K23.0, K23.1, K25-K28, K29.3, K29.4, K29.5, K35-K37, K52.8, K52.9, K61, K63.0, K63.2, K65.0, K67.8, K90.8, K93.0, R11
Respiratory infection	A15-A19, A48.1, A48.2, B59, J00-J06, J10-J22, J32, J36, J37, J39.0, J39.1, J40-J42, J43.0, J44.0, J47, J56, J85, J86, J98.8, N74.0, N74.1
Genitourinary infection	N30.0, N34.1, N35.1, N37, N39.0, N41.0, N41.1, N41.2, N41.3, N43.1, N45, N48.1, N48.2, N49, N51, N70-N74, N75.1, N76.4, N87
Symptoms and signs	
Abdominal pain	R10-R19, K59
General symptoms and signs	R50-R69
Respiratory and circulatory symptoms	R00-R09
Other symptoms	R20-49, R70-R99
Other conditions	
Non-inflammatory disease of the female genitourinary system	N80-N98

Other joint/soft tissue disorders	M20-M25, M70-M79
Diseases of male genital organs	N40-N51

Codes for long-term conditions (apart from sickle cell disease and inflammatory bowel disease) were taken from *Hardelid et al.*[1], codes for infections were taken from *Baker et al.*[2]. Other groups are based on ICD-10 subchapters.

1. Hardelid P, Dattani N, Davey J, et al. Overview of child deaths in the four UK countries. HQIP bit.ly/hardelidreport
2. Barker MG, Barnard LT, Kvalsvig A, et al. Increasing incidence of serious infectious diseases and inequalities in New Zealand: a national epidemiological study. *The Lancet* 2012;**379**:1112-9.

For peer review only

Appendix C: Sensitivity analyses

In Figure A1 in Appendix A, we showed the overall admission rate for children and young people aged 10-24 years old. Here we show the results, as they would have been in Table 1, if we had included all emergency admission, compared to the results presented in the paper (excluding injury admissions, and maternity/pregnancy related emergency admissions for females, Table C1).

Table C1: Sensitivity analysis of exclusion criteria on rates and trends in emergency admission rates before, during and transition from pediatric to adult care

	Proportion of admissions 10-15 years	Average rate 10-15 years	Average rate 19-24 years		Trend age 10-15 years	Trend age 16-18 years	Trend age 19-25 years
**FEMALES**	Rate per 1,000 person-years			IRR[†] (99% CI)	Annual change in admission rate per 1,000 py		
Overall	*100*	39.4	81.6	2.07 (2.06 – 2.08)	↑3.61	↑2.86	↘-0.78
Overall, excluding injury/maternity admissions	82.4	32.5	52.7	1.62 (1.61 – 1.63)	↑4.48	↑3.78	↓-1.39
Injury admissions	16.7	6.6	6.9	1.05 (1.03-1.06)	→0.10	↗0.41	↘-0.31
Maternity/pregnancy admissions	0.9	0.4	22.2	61.9 (61.8 – 62.0	↗0.26	↑4.62	↑1.34
**MALES**							
Overall	*100*	41.0	50.8	1.24 (1.23 – 1.25)	↗0.27	↑1.91	↗0.30
Overall, excluding injury admissions	67.0	27.5	34.6	1.26 (1.25 – 1.27)	↘-0.70	↑2.80	→0.07
Injury admissions	33.0	13.5	16.2	1.20 (1.19 – 1.21)	↗0.94	↑1.49	↘-0.52

*Excluding maternity/pregnancy related admissions and injury admissions
†Incidence rate ratio comparing emergency admission rates before and after transition

By excluding injury and maternity/pregnancy related emergency admissions, we have slightly underestimated the increase in emergency admission rates across transition for both males and females.

For peer review only

For peer review only - http://bmjopen.bmj.com/site/about/guidelines.xhtml

In the manuscript, we assessed children with underlying long-term conditions as a separate group. We assessed whether children and young people had a code indicating a LTC recorded in any admission record (emergency or elective) in the previous 5 years. As a sensitivity analysis, we explored the effects of only using admission records from the last year or last 3 years (Table C2).

Table C2: Sensitivity analysis of the effect of including long-term conditions (LTCs) recorded in the last year, last 3 years, or last 5 years

	Proportion of admissions 10-15 years	Average rate 10-15 years	Average rate 19-24 years		Trend age 10-15 years	Trend age 16-18 years	Trend age 19-25 years
**FEMALES***	Rate per 1,000 person-years			IRR† (99% CI)	Annual change in admission rate per 1,000 py		
No LTCs	41.8	13.6	17.1	1.26 (1.25 – 1.28)	↗0.87	↗0.92	↘-0.61
LTC recorded in previous year	56.0	18.2	33.3	1.83 (1.82-1.84)	↑3.49	↑2.54	↘-0.77
LTC recorded in previous 3 years	57.5	18.7	34.7	1.86 (1.85-1.87)	↑3.58	↑2.71	↘-0.79
LTC recorded in previous 5 years	58.2	18.9	35.5	1.88 (1.87-1.89)	↑3.61	↑2.86	↘-0.78
**MALES***							
No LTCs	45.3	12.4	12.0	0.96 (0.95-0.98)	↘-0.97	↗0.88	↘-0.23
LTC recorded in previous year	52.6	14.4	21.7	1.50 (1.49 – 1.51)	↗0.30	↑1.84	↗0.26
LTC recorded in previous 3 years	55.8	15.3	22.3	1.45 (1.44 – 1.47)	↗0.29	↑1.90	↗0.28
LTC recorded in previous 5 years	56.0	15.4	22.6	1.47 (1.46 – 1.48)	↗0.27	↑1.91	↗0.30

*Excluding maternity/pregnancy related admissions and injury admissions
†Incidence rate ratio comparing emergency admission rates before and after transition

When using records for the previous five years, we find slightly higher emergency admission rates for children with underlying long-term conditions compared to looking back just one or three years. However, the relatively small differences indicate most LTCs were recorded in the year prior to the index emergency admission.

For peer review only

STROBE 2007 (v4) Statement—Checklist of items that should be included in reports of *cross-sectional studies*

Section/Topic	Item #	Recommendation	Reported on page #
**Title and abstract**	1	(*a*) Indicate the study's design with a commonly used term in the title or the abstract	1
		(*b*) Provide in the abstract an informative and balanced summary of what was done and what was found	2
**Introduction**			
Background/rationale	2	Explain the scientific background and rationale for the investigation being reported	3-4
Objectives	3	State specific objectives, including any prespecified hypotheses	4
**Methods**			
Study design	4	Present key elements of study design early in the paper	5
Setting	5	Describe the setting, locations, and relevant dates, including periods of recruitment, exposure, follow-up, and data collection	
Participants	6	(*a*) Give the eligibility criteria, and the sources and methods of selection of participants	5
Variables	7	Clearly define all outcomes, exposures, predictors, potential confounders, and effect modifiers. Give diagnostic criteria, if applicable	6
Data sources/ measurement	8*	For each variable of interest, give sources of data and details of methods of assessment (measurement). Describe comparability of assessment methods if there is more than one group	5-6
Bias	9	Describe any efforts to address potential sources of bias	6-7
Study size	10	Explain how the study size was arrived at	5
Quantitative variables	11	Explain how quantitative variables were handled in the analyses. If applicable, describe which groupings were chosen and why	6-7
Statistical methods	12	(*a*) Describe all statistical methods, including those used to control for confounding	6-7
		(*b*) Describe any methods used to examine subgroups and interactions	7
		(*c*) Explain how missing data were addressed	7
		(*d*) If applicable, describe analytical methods taking account of sampling strategy	n/a
		(*e*) Describe any sensitivity analyses	7
**Results**			

For peer review only

Participants	13*	(a) Report numbers of individuals at each stage of study—eg numbers potentially eligible, examined for eligibility, confirmed eligible, included in the study, completing follow-up, and analysed	7
		(b) Give reasons for non-participation at each stage	n/a
		(c) Consider use of a flow diagram	n/a
Descriptive data	14*	(a) Give characteristics of study participants (eg demographic, clinical, social) and information on exposures and potential confounders	7-8 (+ appendix A)
		(b) Indicate number of participants with missing data for each variable of interest	7-8
Outcome data	15*	Report numbers of outcome events or summary measures	7-8
Main results	16	(*a*) Give unadjusted estimates and, if applicable, confounder-adjusted estimates and their precision (eg, 95% confidence interval). Make clear which confounders were adjusted for and why they were included	8-10
		(*b*) Report category boundaries when continuous variables were categorized	9-10
		(*c*) If relevant, consider translating estimates of relative risk into absolute risk for a meaningful time period	10
Other analyses	17	Report other analyses done—eg analyses of subgroups and interactions, and sensitivity analyses	10-11 (+ appendix C)
**Discussion**			
Key results	18	Summarise key results with reference to study objectives	11
Limitations	19	Discuss limitations of the study, taking into account sources of potential bias or imprecision. Discuss both direction and magnitude of any potential bias	12-13
Interpretation	20	Give a cautious overall interpretation of results considering objectives, limitations, multiplicity of analyses, results from similar studies, and other relevant evidence	13-14
Generalisability	21	Discuss the generalisability (external validity) of the study results	11-12
**Other information**			
Funding	22	Give the source of funding and the role of the funders for the present study and, if applicable, for the original study on which the present article is based	16

*Give information separately for cases and controls in case-control studies and, if applicable, for exposed and unexposed groups in cohort and cross-sectional studies.

Note: An Explanation and Elaboration article discusses each checklist item and gives methodological background and published examples of transparent reporting. The STROBE checklist is best used in conjunction with this article (freely available on the Web sites of PLoS Medicine at http://www.plosmedicine.org/, Annals of Internal Medicine at http://www.annals.org/, and Epidemiology at http://www.epidem.com/). Information on the STROBE Initiative is available at www.strobe-statement.org.

For peer review only

BMJ Open

Emergency admissions and long-term conditions during transition from paediatric to adult care: a cross-sectional study using Hospital Episode Statistics data

Journal:	*BMJ Open*
Manuscript ID	bmjopen-2017-021015.R1
Article Type:	Research
Date Submitted by the Author:	19-Apr-2018
Complete List of Authors:	Wijlaars, Linda; UCL, Population, Policy and Practice; University College London, Administrative Data Research Centre for England<br>Hardelid, Pia; UCL Institute of Child Health, Centre for Paediatric Epidemiology and Biostatistics<br>Guttmann, Astrid; Institute for Clinical Evaluative Sciences, ; The Hospital for Sick Children, Division of Pediatric Medicine<br>Gilbert, Ruth; UCL Institute of Child Health, Centre for Paediatric Epidemiology and Biostatistics
**Primary Subject Heading**:	Epidemiology
Secondary Subject Heading:	Health informatics, Paediatrics
Keywords:	PAEDIATRICS, Chronic conditions, Administrative hospital data, MENTAL HEALTH, Emergency admissions, Transition from paediatric to adult care

SCHOLARONE™
Manuscripts

Emergency admissions and long-term conditions during transition from paediatric to adult care: a cross-sectional study using Hospital Episode Statistics data

Linda PMM Wijlaars [a,b] (linda.wijlaars@ucl.ac.uk), Pia Hardelid[a] (p.hardelid@ucl.ac.uk), Astrid Guttmann[c] (astrid.guttmann@ices.on.ca), Ruth Gilbert[a,b] (r.gilbert@ucl.ac.uk)

Affiliations:

[a]Population, Policy and Practice / Children's Policy Research Unit, University College London Great Ormond Street Institute of Child Health, 30 Guilford Street, WC1X 9ED London, UK;

[b]Administrative Data Research Centre for England, University College London, 222 Euston Road, NW1 2DA London, United Kingdom,

[c]Institute of Clinical Evaluation Sciences, Toronto, Ontario, Canada

Corresponding author: Linda Wijlaars

Short title: Emergency admissions during transition

Keywords: Administrative data, transition, paediatrics, chronic conditions, mental health, hospitalizations

Abstract word count: 274

Article word count: 3295

For peer review only

Abstract:

Objectives: To determine whether changes in emergency admission rates during transition from paediatric to adult hospital services differed in children and young people (CYP) with and without underlying long-term conditions (LTCs).

Design: Cross-sectional study

Setting: Emergency admissions between 2009 and 2011 recorded in Hospital Episode Statistics Admitted Patient Care data in England.

Participants: 763,199 CYP aged 10-24 years with and without underlying LTC (LTCs were defined using ICD-10 codes recorded in the past five years).

Primary and secondary outcome measures: We calculated emergency admission rates before (10-15 years), and after transition (19-24 years) stratified by gender, LTC, and primary diagnosis. We used negative binomial regression to estimate adjusted incidence rate ratios.

Results: We included 1,109,978 emergency admissions, of which 63.2% were in children with LTCs. The emergency admission rate increased across the age of transition for all CYP, more so for those with LTCs (IRR_{LTC}: 1.55, 99% CI: 1.47-1.63), compared to those without (IRR_{noLTC}: 1.21, 99% CI: 1.18-1.23).

Rates increased most rapidly for CYP with mental health problems, MEDReG (metabolic/endocrine/digestive/renal/genitourinary) disorders, and multiple LTCs (both genders), and respiratory disorders (females only). Small or no increased rates were found for CYP without LTCs and for those with cancer or cardiovascular disease. Increases in length of stay were driven by long admissions (10+ days) for a minority (1%) of CYP with mental health problems and potentially psychosomatic symptoms. Non-specific symptoms related to abdominal pain (girls only), gastrointestinal and respiratory problems were the most frequent primary diagnoses.

For peer review only - http://bmjopen.bmj.com/site/about/guidelines.xhtml

Conclusions: The increased rates and duration of emergency admissions and predominance of non-specific admission diagnoses during transition in CYP with underlying LTCs, may reflect unmet physical or mental health needs.

Strengths and limitations of this study

- This study included all hospital admissions to the NHS in England over three years, minimising selection bias and allowing for comparison of patients using paediatric and adult services.
- Our analysis used a standardised and validated coding system to identify underlying long-term conditions, based on any diagnostic codes recorded at the current or past admissions in the previous five years.
- As we used a cross-sectional design with admissions as the denominator, we did not account for individuals with multiple admissions (i.e. clustering) and could have overestimated the contribution of certain conditions that are associated with multiple emergency admissions.
- We may have included young people who were first diagnosed with long-term conditions during or after the age of transition.

For peer review only

Background

Transition from paediatric to adult services presents challenges to children and young people (CYP), their families and to service providers.[1] Firstly, the team managing the CYP's condition often shifts from specialist, hospital-based to generic, community-based care by general practitioners (GPs). For instance, for diabetes care in the UK, care for children is coordinated by paediatric endocrinologists in hospital, while diabetes care for adults is led by primary care.[2] Additionally, as the clinicians and thresholds for access to specialist care change with the shift to adult services ongoing healthcare is often disrupted. This can be

For peer review only - http://bmjopen.bmj.com/site/about/guidelines.xhtml

particularly problematic for children with long-term conditions (LTCs), who account for 41% of all emergency admission in children in England,[3] as they often have little contact with their primary care physician prior to transition.[4]

Secondly, transition to adult care occurs during a critical and vulnerable period for children with LTCs. Adult services often expect CYP to assume responsibility for their own care despite the fact that psychological, social and physiologic changes associated with adolescence can disrupt self-management of LTCs.[5,6] Finally, the type of clinical problems experienced by CYP during transition are changing due to the increased survival of young children with complex or multiple LTCs partly due to improved care and support for (very) pre-term infants and for CYP with conditions that would previously have been lethal in early childhood.[7,8]

We have previously reported that rates of emergency and elective admissions increase in young people around the time of transition to adult services.[9] Most studies on care across the transition to adult care have used qualitative methods to evaluate patient or clinician expectations and experience,[10] or have studied outcomes in a relatively small, regionally defined or disease specific population.[11,12] Our primary aim was to use cross-sectional analyses of hospital administrative data for England to compare changes in rates and duration of emergency admission to hospital for CYP with and without underlying LTCs before and after the period of transition from paediatric to adult care. In secondary analyses, we explored changes for LTC groups and determined which primary diagnostic groups accounted for changes in admission rates.

For peer review only - http://bmjopen.bmj.com/site/about/guidelines.xhtml

Methods

Data source

We used anonymised patient-level Hospital Episode Statistics (HES) data for admissions to English National Health Service (NHS) hospitals from 1 April 2009 to 31 March 2012.[13] We used three years of data to ensure any patterns were not due to single year fluctuations. We identified admissions as continuous periods in hospital that could consist of several finished consultant episodes (FCEs – a period of hospital stay under a single consultant).[14] Admissions that occurred within a day of each other or admissions that included a hospital transfer were considered as a single admission.

Population

We included adolescents and young people aged 10 to 24 years in our analysis in order to determine incidence rates before (ages 10-15 years) and after (19-24 years) transition. We defined transition from paediatric to adult healthcare between the ages of 16 to 18 year as in practice, the exact age of transition in the NHS can vary between different NHS services and/or conditions.[15–18]

Our main outcome measure was the incidence rate ratio (IRR) of emergency admissions after compared with before the age of transition. We considered emergency (or unplanned) admission to be a clinically important indicator of ill-health, which might be affected by the quality of health care received from hospital or care in the community during transition. We excluded emergency injury admissions from the primary outcome as these would be strongly affected by personal factors or public health or education interventions (e.g. reaching the legal driving and legal drinking age)[9,19] However, injury admissions due to intentional self-harm (identified by a second diagnosis code indicating self-harm[20]) were included as these could signify an underlying mental health problem. We further excluded admissions

For peer review only - http://bmjopen.bmj.com/site/about/guidelines.xhtml

related to pregnancy and maternities as we expected these to increase with age unrelated to transition from paediatric to adult services.

Variable definitions

We identified CYP with and without underlying LTCs using validated *International Classification of Diseases, 10th Revision* (ICD-10) codes[14] recorded in any diagnostic code during the index admission or any hospital admission in the previous five years. We defined nine LTC groups: 1) Mental health, 2) Cancer and blood disorders, 3) Chronic infections, 4) Respiratory disorders, 5) Metabolic, endocrine, digestive, renal, genitourinary disorders (MEDReG), 6) Musculoskeletal and skin disorders, 7) Neurological disorders, 8) Cardiovascular disorders, and 9) Multiple LTCs (for children with LTCs from more than one group).

Other characteristics included as potential confounders were ethnicity, quintile of deprivation and whether the CYP was referred by a GP or Emergency Department. Deprivation was based on postcode of residence at admission and measured using the Index of Multiple Deprivation 2004 (IMD).[21] Analyses were stratified by sex.

We used the primary diagnosis at admission to define the main reason for admission grouped as: LTCs (only as primary diagnosis), infection, signs and symptoms, and other (see Appendix A, eTable A1). We calculated length of stay by subtracting the discharge date from the admission date and classified admissions as day cases if admission and discharge occurred on the same date.

Statistical analyses

As this is an exploratory study, we used a cross-sectional design. We calculated incidence rates for the years 2009-2011 per 1,000 person-years by sex, using the number of admissions for each calendar year and age group divided by the mid-year population estimates as the

For peer review only - http://bmjopen.bmj.com/site/about/guidelines.xhtml

denominator population from the Office of National Statistics[22].The primary analysis determined the change in the incidence of emergency inpatient admissions measured by the incidence rate ratio (IRR) for the average incidence after transition (19-24 years) divided by the average incidence before transition (10-15 years).

We determined whether the IRR varied significantly according to whether a child had an underlying LTC recorded in the past five years, as well as by primary diagnosis at admission. To correct these estimates for confounding, we used a negative binomial regression model (to correct for overdispersion) with admission counts as the outcome variable, age, underlying LTC, sex, deprivation quintile and ethnicity as explanatory variables and population size as the offset. We used linear combination of regression coefficients to describe the IRRs for subgroups. To determine the effect of long-term conditions on the increase in admission rates across transition we calculated the population-attributable fraction (PAF).[23]

We conducted sensitivity analyses to assess whether results changed when including admissions related to injury and maternity. In addition, we assessed the effect of changing the criterion for underlying LTCs from being recorded in the previous 5 years to 3 or 1 years.

All analyses were stratified by sex and performed in Stata SE version 13.0 (Statacorp, College Station, Texas, US). Figures were produced using Microsoft Excel.

Patient and Public Involvement

Patients were not involved in the design of this study. In order to disseminate our results to young people, we worked with the National Children's Bureau's Young Research Advisors group to develop a short animation (https://youtu.be/u_NG0Vb3tec) that highlights the results they found most important and explore future research priorities.

For peer review only

Results

Overall

We analysed 1,109,978 emergency admissions in 763,199 CYP aged 10-24 years. 680,322 (57.3%) admissions occurred in females. Overall, 701,834 (63.2%) admissions occurred in CYP with a record of a LTC in the last 5 years (eTable B1 in Appendix B). This proportion increased from 57.2% in 10-15 year olds to 66.6% in 19-24 year olds.

The increase in the total rate of emergency admissions (excluding elective admissions and emergency admissions for injury and pregnancy-related problems, eFigure B1 in Appendix B) was higher in females than in males: incidence rate ratios comparing average rates after with before transition were 1.64 (99% CI: 1.63-1.64) for females and 1.17 (99% CI: 1.16-1.18) for males. The increase in rates of emergency admission in both sexes occurred predominantly in CYP with underlying LTCs (Figure 1). In the fully adjusted model (Table 1), transition was associated with a modest increase in emergency admission rates for children without LTCs (IRR: 1.21, 99% CI: 1.18-1.23). However, for CYP with LTCs, the effect of transition was stronger (IRR: 1.55, 99% CI: 1.47-1.63).

If CYP with LTCs had the same increase in rates of emergency admission across transition as CYP without LTCs the overall increase in admissions would decrease by 26.3% (population attributable factor (PAF), 99% CI: 25.9% – 26.7%) among females and 25.7% (99% CI: 25.2%– 26.1%) among males.

Inequalities increased across transition (Figure 2). Emergency admission rates varied from 2.2 to 3.6 per 1,000py from most affluent to most deprived quintiles when children were aged 10 years, but this gap increased to 3.8 to 7.0 for 24 year olds.

Admission rates by specific underlying long term condition

For peer review only - http://bmjopen.bmj.com/site/about/guidelines.xhtml

For peer review only

The steepest increase in emergency admission rates occurred in CYP who had records indicating underlying mental health problems (Figure 3 eTable B2 in Appendix B). Mental health problems were recorded in 31.2% of emergency admissions in 16-18 year olds, predominantly disorders related to alcohol/drug use, self-harm, depression, eating disorders and schizophrenia. CYP with records indicating MEDReG disorders or multiple LTCs also experienced large increases in admission rates across transition. Emergency admissions for cancer and blood disorders and cardiovascular conditions remained stable or decreased across transition.

Specific underlying LTCs of asthma, diabetes, and epilepsy increased only marginally, though more so in females (Figures 3 and 4). As expected, larger increases were seen for inflammatory bowel disease (IBD), as this condition often emerges during adolescence. Admissions of CYP with these underlying LTCs (asthma, diabetes, epilepsy or IBD) contributed to a minority of admissions before (14.8%) and after (11.3%) transition. Emergency admissions for these four conditions accounted for 14.1% (PAF 99% CI: 13.8%-14.6%) of the increase across transition, just over half of the total effect (26.0%).

Primary diagnosis on admissions

Although admissions increased in CYP with underling LTCs, the primary reason for admission was often not the LTC itself. We found only modest increases in rates of emergency admission during transition with primary diagnoses recorded for specific LTCs. (Figure 3, eTable B3 in Appendix B). Admissions with a primary diagnosis of asthma or epilepsy remained relatively stable across transition (Figure 4) while IBD increased.

For peer review only - http://bmjopen.bmj.com/site/about/guidelines.xhtml

As primary diagnoses for LTCs did not explain the increase in emergency admission rates (eTable B4 in Appendix B), we explored other reasons for admissions. Emergency admissions with primary diagnoses for "signs and symptoms" saw strong increases over the transition. Admissions for abdominal pain, circulatory and respiratory symptoms, and general symptoms all increased substantially. Abdominal pain in particular was the primary reason for emergency admission in 19.7% (for females) and 9.8% (for males) at ages 16-18 years. Overall, emergency admission rates for symptoms increased for both females (IRR: 1.83 (99% CI: 1.81-1.84) and males (IRR: 1.21 (99% CI: 1.20-1.23), eTable B3 in Appendix B).

Emergency admissions for infections also increased (IRR: 1.72 for females, 1.29 for males, eTable B3 in Appendix B). There were also increases in 'any other' diagnoses for emergency admissions (where the primary diagnosis was not a LTC, infection nor symptom), although admission rates for individual reasons were low (eTable B3 in Appendix B).

Duration of admission

The length of stay for emergency admissions increased with age from an average of 2.2 days at age 10 years to 4.5 days at age 24 years for females, and from 1.9 to 9.2 for males (Figure 5). The median length of stay was 1 day for the entire age range, for both males and females. While day cases were the most common type of admission at age 10 years (38% for females and 41% for males), there was a drop in the proportion of day cases around the age of transition (age 16-18 years) to 27% for females and 28% for males.

A small proportion of females and males had very long admissions (10 days or longer, n=84,812, 7.1% of total emergency admissions), which increased from 5% before to 7% after transition for females and 4% to 11% for males. A large proportion (18,812, 22.3%) of these long-stay admissions were related to mental health problems such as schizophrenia and

For peer review only - http://bmjopen.bmj.com/site/about/guidelines.xhtml

personality disorders (eTable B5 in Appendix B), and a minority (4,358, 5.2%) had an unspecified primary diagnosis (ICD-10 code R69.X).

Young adults were more likely to be admitted via the emergency department rather than via their primary care physician or other methods: the proportion of emergency admission via ED increased for females from 51% before transition to 69% after transition and form 53% to 71% for males.

Sensitivity analyses

The results of the sensitivity analyses (Appendix C) show that by excluding injury and maternity/pregnancy related admissions (eTable C1) we have underestimated the overall increase in emergency admission rates for females (26%) and males (6%). However, the higher rate of admission in females was entirely due to maternity-related emergency admissions which increased from 0.4 to 22.2 per 1,000 person-years.

IRRs for emergency admissions before and after transition for CYP affected by underlying LTCs decreased slightly (from 1.88, 99% CI: 1.87-1.89 to 1.83, 99% CI: 1.82-1.84) when we changed our definition from LTCs recorded in the previous 5 years, to 3 or 1 year (eTable C2).

Discussion

Emergency admission rates increased at the age when young people transition from paediatric to adult healthcare. The increase in emergency admission rates after the age of transition was significantly higher for those with underlying LTCs, compared to CYP without LTCs. Although emergency admissions increased in CYP with underlying LTCs (most steeply for mental health problems, present in 31% of included emergency admissions), the primary diagnosis on admission was often for other problems, most commonly signs and symptoms,

For peer review only

and infections. Increases were more marked for females than for males, and for the most deprived CYP.

Average length of stay also increased after transition, particularly for males. A substantial minority of the emergency admissions with long lengths of stay (10+ days) were for mental health conditions: schizophrenia and psychoses for males, and eating and personality disorders for females.

Context

Our results are similar to findings from a study in Ontario, Canada, that found an increase in rates of emergency admissions for young people with diabetes during the transition from paediatric to adult services[12]. The rates for diabetes mellitus-related admissions were lower in our population (1-3 per 1,000 vs. 7-10 per 100 patient-years in Ontario) possibly due to differences in the population and in how conditions are coded. However, we found similar trends with regards to deprivation and female gender.

We found that while emergency admission rates for CYP with LTC increased across transition for most sub-groups, rates remained stable for CYP with cardiovascular disorders and cancer or blood disorders. This finding may reflect the intensity of paediatric and adult specialist care for these conditions and a reduced likelihood of emergency admission because other types of hospital care (e.g. clinic review or planned admission) can be readily accessed. Alternatively, transition from paediatric services may be deferred.[24,25] For example, paediatric services for cancer can be commissioned up to age 24 years. A further explanation could be improved transition, e.g. congenital heart disease services have reported using transition nurses to provide continuity between paediatric and adult services [18,25].

Emergency admission rates were more likely to increase in children from more deprived backgrounds, although the effect size was small. We found that after transition emergency

For peer review only - http://bmjopen.bmj.com/site/about/guidelines.xhtml

admission rates stabilized, both overall and for many LTC groups by age 24 years. Also taking the drop in the proportion of day cases and increase in length of stay into account, this could be explained by a higher admission threshold for adults and potentially unmet health needs as a result.

Strengths and limitations

By analysing all NHS admissions in England, we avoided bias due to different referral pathways for CYP as they transition to adult services. Our analysis of underlying LTCs, based on any diagnostic codes recorded at the current or past admissions in the previous five years, showed that CYP with LTCs often present with primary diagnoses related to symptoms or conditions other than their LTC. Previous studies have focused on well-recognized LTCs such as asthma, diabetes, epilepsy and IBD[26] but in our analyses, these conditions accounted for only half of the increase in admissions, and a minority of primary diagnoses. Our finding of relative stability in admissions for cancer and cardiovascular conditions suggests that care over the transition differs between conditions.

The cross-sectional study design of our study is a limitation. As we analysed admissions without taking into account that individuals could have multiple admissions (i.e. clustering) we might have overestimated the contribution of certain conditions that are associated with multiple emergency admissions. Additionally, we may have included young people who were first diagnosed with LTCs after the age of transition.

We used a retrospective capture of past admissions to categorize CYP's emergency admissions into underlying LTC or no LTC. Our cross-sectional analyses of the whole population complement longitudinal analyses that follow individual CYP with and without LTCs across the period of transition. However, longitudinal analyses require identification of inception points, such as first diagnosis of the long-term condition. This is challenging using

For peer review only - http://bmjopen.bmj.com/site/about/guidelines.xhtml

hospitalization data as many CYP with underlying LTCs such as asthma are mostly managed in primary care. Such analyses would be feasible in future studies using linked primary care and hospitalization data for whole populations.

Implications

Emergency admission may be a manifestation of the widely recognized difficulties young people face when they transition to adult health services.[1,18,27] Emergency admissions are more common and longer after transition, resulting in higher healthcare costs. Our findings of disparities according to presence of underlying LTC and deprivation suggest potential to reduce emergency admissions across transition by focusing on young people with underlying LTCs. This will require system-level strategies and targeted investments.[28]

The high rates of emergency admissions for symptoms among CYP with LTCs may reflect a common preoccupation among adolescents with 'normal' physical symptoms, which generates more clinical concern and need for investigation in CYP with LTCs.[29,30] Adult care often involves less routine review or anticipatory management of problems and has been reported to be more focused on disease management rather than the CYP's broader health and psychosocial needs.[18] Alternatively, symptoms may reflect undiagnosed complications or morbidity or maybe a manifestation of mental health distress, which might not be investigated in the context of an emergency hospital admission.

A potential unmet need for mental health care is borne out by the steep increase in emergency admission with underlying mental health problems across transition. Previous research has claimed that UK mental health services around transition are not fit for purpose[31] and has called for prioritization of initiatives and research to improve transition care.

Primary care is the first line provider of mental health care for adolescents and the main provider for adults in England and elsewhere. However, rates of GP consultations decline

For peer review only - http://bmjopen.bmj.com/site/about/guidelines.xhtml

steeply during adolescence, particularly among males[32], also reflected in our study by the decreasing proportion of admissions in older age groups that were referred by the GP. To improve provision of care during transition, services in primary and community care need to be made more attractive and accessible to adolescents to enable improved (mental) health care and thereby reduce the risk of admissions.[33–35] However, although there have been calls to improve primary care involvement in transition care, there is little evidence on the most effective ways to do this.[36] Further research into regional variation could help identify existing intervention or programmes with better outcomes for young people transitioning to adult care.

Hospital specialties in the UK are also making changes to transitional care. Several practice guidelines focus on early planning and communication, and having a single named practitioner to coordinate transition care and support.[4,18,27,37] Our findings suggest that future healthcare planning needs to shift from management of transition for specific LTCs to transitional care for the whole person that meets their holistic care needs, particularly mental health, thereby averting serious acute problems that require admission[38].

Abbreviations:

CI – Confidence Interval;
CYP – Children and Young People;
DM – Diabetes Mellitus;
ED – Emergency Department;
FCE – Finished Consultant Episode;
GP – General Practitioner;
HES – Hospital Episode Statistics;
ICD-10 – International Statistical Classification of Diseases and Related Health Problems 10th Revision;
IMD – Index of Multiple Deprivation;
LTCs – Long Term Conditions;
MEDReG disorders – Metabolic, Endocrine, Digestive, Renal or Genitourinary disorders;
NHS – (English) National Health Service;
py – person-year;
RR – Rate Ratio

For peer review only - http://bmjopen.bmj.com/site/about/guidelines.xhtml

Funding statement
Dr Wijlaars is funded by the Department of Health Policy Research Programme through funding to the Policy Research Unit in the Health of Children, Young People and Families (grant reference number 109/0001). Dr Hardelid is funded by a National Institute for Health Research postdoctoral fellowship (number PDF-2013-06-004). This article represents independent research funded by the National Institute for Health Research (NIHR) and the Department of Health. The views expressed in this publication are those of the author(s) and not necessarily those of the NHS, the National Institute for Health Research or the Department of Health.
Prof Guttmann is funded by a Canadian Institutes for Health Research Applied Chair Award in Reproductive and Child Health Services Research.
We also acknowledge the support from the Farr Institute of Health Informatics Research (MRC Grant Nos: London MR/ K006584/1).

Competing interests
The authors declare that they have no competing interests.

Authors contributions
Linda Wijlaars: Dr Wijlaars conceptualized and designed the study, carried out the analyses, drafted the initial manuscript, and approved the final manuscript as submitted.

Pia Hardelid: Dr Hardelid helped design the study, reviewed and revised the manuscript, and approved the final manuscript as submitted.

Astrid Guttmann: Prof Guttmann helped conceptualize the study, critically reviewed the manuscript, and approved the final manuscript as submitted.

Ruth Gilbert: Prof Gilbert helped conceptualize and design the study, reviewed and revised the manuscript, and approved the final manuscript as submitted.

All authors approved the final manuscript as submitted and agree to be accountable for all aspects of the work.

Ethics approval
Use of Hospital Episodes Statistics data was approved by the Health and Social Care Information Centre for the purpose of this study (DARS-NIC-393510-D6H1D-v1.11).

Source data can be accessed by researchers applying to the Health and Social Care Information Centre for England. Copyright © 2017, Re-used with the permission of the Health and Social Care Information Centre. All rights reserved.

Data sharing statement
Technical appendix with details on code list used to identify primary diagnoses group is available in appendix A. Statistical code is available upon request from authors.

For peer review only - http://bmjopen.bmj.com/site/about/guidelines.xhtml

Acknowledgements
We would like to thank Jenny Woodman and David Cottrell for their input into the writing and amending of this manuscript, as well as members of the Policy Research Unit in the Health of Children, Young People and Families: Catherine Law, Russell Viner, Miranda Wolpert, Amanda Edwards, Steve Morris, Helen Roberts, Terence Stephenson, and Cathy Street.

References

1 Crowley R, Wolfe I, Lock K, *et al.* Improving the transition between paediatric and adult healthcare: a systematic review. *Arch Dis Child* 2011;:archdischild202473. doi:10.1136/adc.2010.202473

2 NICE. NG18: Diabetes (type 1 and type 2) in children and young people: diagnosis and management. 2015.http://www.nice.org.uk/guidance/ng18 (accessed 16 Nov 2015).

3 Wijlaars LP, Hardelid P, Woodman J, *et al.* Contribution of recurrent admissions in children and young people to emergency hospital admissions: retrospective cohort analysis of hospital episode statistics. *Arch Dis Child* 2015;:archdischild-2014-307771. doi:10.1136/archdischild-2014-307771

4 Contact a Family. Putting families with disabled children at the heart of the NHS reforms in England (2011). 2012.

5 Suris J-C, Michaud P-A, Viner R. The adolescent with a chronic condition. Part I: developmental issues. *Arch Dis Child* 2004;**89**:938–42. doi:10.1136/adc.2003.045369

6 Sawyer SM, Drew S, Yeo MS, *et al.* Adolescents with a chronic condition: challenges living, challenges treating. *The Lancet* 2007;**369**:1481–9. doi:10.1016/S0140-6736(07)60370-5

7 Cohen E, Kuo DZ, Agrawal R, *et al.* Children with medical complexity: an emerging population for clinical and research initiatives. *Pediatrics* 2011;**127**:529–38. doi:10.1542/peds.2010-0910

8 Larroque B, Bréart G, Kaminski M, *et al.* Survival of very preterm infants: Epipage, a population based cohort study. *Arch Dis Child - Fetal Neonatal Ed* 2004;**89**:F139–44. doi:10.1136/adc.2002.020396

9 Wijlaars L, Hardelid P, Woodman J, *et al.* Contribution of recurrent admissions in children and young people to emergency hospital admissions: retrospective cohort analysis of hospital episode statistics. *Arch Dis Child* in press.

10 Scal P, Ireland M. Addressing Transition to Adult Health Care for Adolescents With Special Health Care Needs. *Pediatrics* 2005;**115**:1607–12. doi:10.1542/peds.2004-0458

11 Reid GJ, Irvine MJ, McCrindle BW, *et al.* Prevalence and Correlates of Successful Transfer From Pediatric to Adult Health Care Among a Cohort of Young Adults With Complex Congenital Heart Defects. *Pediatrics* 2004;**113**:e197–205.

For peer review only - http://bmjopen.bmj.com/site/about/guidelines.xhtml

12 Nakhla M, Daneman D, To T, *et al.* Transition to Adult Care for Youths With Diabetes Mellitus: Findings From a Universal Health Care System. *Pediatrics* 2009;**124**:e1134–41. doi:10.1542/peds.2009-0041

13 Health and Social Care Information Centre 1 Trevelyan Square. Hospital Episode Statistics. 2012.http://www.hscic.gov.uk/hes (accessed 28 Aug 2014).

14 Hardelid P, Dattani N, Davey J, *et al.* Overview of child deaths in the four UK countries. RCPCH 2013. bit.ly/hardelidreport

15 Kennedy I. Getting it right for children and young people: overcoming cultural barriers in the NHS so as to meet their needs. Department of Health 2010. https://www.gov.uk/government/publications/getting-it-right-for-children-and-young-people-overcoming-cultural-barriers-in-the-nhs-so-as-to-meet-their-needs

16 Barnes D, Wistow R, Dean R, *et al.* National child and adolescent mental health service mapping exercise 2005. Durham University, School ofApplied Social Sciences 2006. http://dro.dur.ac.uk/4333/1/4333.pdf?DDD34+dss4ae+dul0jk

17 Davies SC. Prevention pays - our children deserve beter. 2013. https://www.gov.uk/government/news/chief-medical-officer-prevention-pays-our-children-deserve-better

18 Care Quality Commission. From the pond into the sea: Children's transition to adult health services. 2014. https://admin.cqc.org.uk/sites/default/files/CQC_Transition%20Report.pdf

19 Gonzalez-Izquierdo A, Cortina-Borja M, Woodman J, *et al.* Maltreatment or violence-related injury in children and adolescents admitted to the NHS: comparison of trends in England and Scotland between 2005 and 2011. *BMJ Open* 2014;**4**:e004474. doi:10.1136/bmjopen-2013-004474

20 Clinical Classifications Service. Chapter XX External Causes of Morbidity and Mortality (V01-Y98). In: *National Clinical Coding Standards ICD-10 5th Edition (2016)*. Leeds: : Health & Social Care Information Centre 2016.

21 Government D for C and L. The English Indices of Deprivation 2004 (revised). 2007.http://webarchive.nationalarchives.gov.uk/20120919132719/http://www.communities.gov.uk/publications/communities/englishindices (accessed 17 Sep 2015).

22 Population Estimates for UK, England and Wales, Scotland and Northern Ireland. Off. Natl. Stat. 2011.http://www.ons.gov.uk/ons/rel/pop-estimate/population-estimates-for-uk--england-and-wales--scotland-and-northern-ireland/population-estimates-timeseries-1971-to-current-year/index.html (accessed 27 Mar 2014).

23 Greenland S, Drescher K. Maximum Likelihood Estimation of the Attributable Fraction from Logistic Models. *Biometrics* 1993;**49**:865–72. doi:10.2307/2532206

24 Cystic Fibrosis Trust. Transition from paediatric to adult care. A guide for commissioners, hospital and clincal team. Bromley: 2013. https://www.cysticfibrosis.org.uk/~/media/documents/life-with-cf/publications/factsheet-transition-to-adult-care-for-commissioners.ashx?la=en

For peer review only - http://bmjopen.bmj.com/site/about/guidelines.xhtml

25 Smith S, Case L, Waterhouse K, *et al.* A blueprint of care for teenagers and young adults with cancer. Teenage Cancer Trust 2012. https://www.teenagecancertrust.org/sites/default/files/Blueprint-of-Care.pdf

26 Cohen E, Gandhi S, Toulany A, *et al.* Health Care Use During Transfer to Adult Care Among Youth With Chronic Conditions. *Pediatrics* 2016;**137**:e20152734. doi:10.1542/peds.2015-2734

27 NICE. NG 43: Transition from children's to adults' services. 2016. https://www.nice.org.uk/guidance/indevelopment/gid-scwave0714 (accessed 16 Nov 2015).

28 Hepburn CM, Cohen E, Bhawra J, *et al.* Health system strategies supporting transition to adult care. *Arch Dis Child* 2015;**100**:559–64. doi:10.1136/archdischild-2014-307320

29 Garber J, Walker LS, Zeman J. Somatization symptoms in a community sample of children and adolescents: Further validation of the Children's Somatization Inventory. *Psychol Assess J Consult Clin Psychol* 1991;**3**:588–95. doi:10.1037/1040-3590.3.4.588

30 Eminson M, Benjamin S, Shortall A, *et al.* Physical Symptoms and Illness Attitudes in Adolescents: An Epidemiological Study. *J Child Psychol Psychiatry* 1996;**37**:519–28. doi:10.1111/j.1469-7610.1996.tb01438.x

31 Memarzia J, Clair MCS, Owens M, *et al.* Adolescents leaving mental health or social care services: predictors of mental health and psychosocial outcomes one year later. *BMC Health Serv Res* 2015;**15**:185. doi:10.1186/s12913-015-0853-9

32 Wang Y, Hunt K, Nazareth I, *et al.* Do men consult less than women? An analysis of routinely collected UK general practice data. *BMJ Open* 2013;**3**:e003320. doi:10.1136/bmjopen-2013-003320

33 Zimmer-Gembeck MJ, Alexander T, Nystrom RJ. Adolescents report their need for and use of health care services. *J Adolesc Health* 1997;**21**:388–99. doi:10.1016/S1054-139X(97)00167-5

34 Klein JD, McNulty M, Flatau CN. Adolescents' access to care: Teenagers' self-reported use of services and perceived access to confidential care. *Arch Pediatr Adolesc Med* 1998;**152**:676–82.

35 Booth ML, Bernard D, Quine S, *et al.* Access to health care among Australian adolescents young people's perspectives and their sociodemographic distribution. *J Adolesc Health* 2004;**34**:97–103. doi:10.1016/j.jadohealth.2003.06.011

36 Bhawra J, Toulany A, Cohen E, *et al.* Primary care interventions to improve transition of youth with chronic health conditions from paediatric to adult healthcare: a systematic review. *BMJ Open* 2016;**6**:e011871. doi:10.1136/bmjopen-2016-011871

37 Rosen DS, Blum RW, Britto M, *et al.* Transition to adult health care for adolescents and young adults with chronic conditions: position paper of the Society for Adolescent Medicine. *J Adolesc Health* 2003;**33**:309–311.

For peer review only - http://bmjopen.bmj.com/site/about/guidelines.xhtml

38 Davis AM, Brown RF, Taylor JL, *et al.* Transition Care for Children With Special Health Care Needs. *Pediatrics* 2014;**134**:900–8. doi:10.1542/peds.2014-1909

For peer review only

For peer review only - http://bmjopen.bmj.com/site/about/guidelines.xhtml

Tables

Table 1: Relative admission rates for CYP with and without LTCs and before (10-15 years) and after (19-24 years) transition

	No LTC	LTC	No LTC	LTC
**Covariate**	crude IRR (99% CI)		adjusted IRR (99% CI)	
Transition	1.18 (1.17-1.18)	1.44 (1.41 – 1.47)	1.21 (1.18 – 1.23)	1.55 (1.47 – 1.63)
Sex (reference = males)	1.07 (1.06 – 1.07)	0.92 (0.90 – 0.94)	1.05 (1.05 – 1.06)	0.98 (0.96 – 1.00)
Age	1.02 (1.01 – 1.02)	1.03 (1.03 – 1.04)	1.00 (0.99 – 1.00)	0.98 (0.98 – 0.99)
IMD '04				
1 - Most deprived	1.10 (1.09 – 1.11)	1.41 (1.36 – 1.46)	1.07 (1.05 – 1.08)	1.17 (1.13 – 1.20)
2	1.07 (1.06 – 1.09)	1.28 (1.23 – 1.32)	1.04 (1.03 – 1.06)	1.08 (1.05 – 1.12)
3	1.07 (1.06 – 1.10)	1.05 (1.01 – 1.09)	1.07 (1.05 – 1.08)	1.02 (0.99 – 1.05)
4	1.02 (1.01 – 1.04)	1.04 (1.00 – 1.07)	1.02 (1.00 – 1.03)	1.02 (0.98 – 1.05)
5 – Least deprived	Reference	Reference	Reference	Reference
Missing	0.88 (0.86 – 0.91)	1.11 (1.01 - 1.22)	0.87 (0.85 – 0.89)	1.01 (0.92 – 1.10)
Ethnicity				
White	Reference	Reference	Reference	Reference
Black	1.21 (1.18 – 1.24)	2.67 (2.56 – 2.79)	1.19 (1.16 – 1.22)	2.49 (2.39 – 2.60)
Asian	1.03 (1.01 – 1.04)	1.07 (1.03 – 1.12)	1.03 (1.01 – 1.04)	1.13 (1.08 – 1.19)
Mixed	0.94 (0.92 – 0.95)	0.93 (0.89 – 0.97)	0.93 (0.92 – 0.95)	0.93 (0.89 – 0.97)
Unknown	0.75 (0.74 – 0.77)	0.43 (0.39 – 0.47)	0.75 (0.74 – 0.77)	0.43 (0.40 – 0.47)
Missing	0.82 (0.80 – 0.84)	0.52 (0.49 – 0.54)	0.82 (0.81 – 0.84)	0.54 (0.51 – 0.57)

IRR = incidence rate ratio, the IRR compares the emergency admission rate before (age 10-15 years) transition with the rate after (age 19-24 years) transition.
Age is included in the model as a continuous variable IRR refers to increase per year of age.
Models are corrected for variables shown in table.

Figures

Figure 1: Emergency and elective admission rates by age and sex
LTCs = long-term conditions as measured in the index admission and admissions in the previous 5 years.
Grey area indicates age range for transition

For peer review only - http://bmjopen.bmj.com/site/about/guidelines.xhtml

Figure 2: Emergency admissions by Index of Multiple Deprivation (IMD04) quintile
Grey area indicates age range for transition

Figure 3: Incidence rate ratios for emergency admissions before (10-15 years) and after (19-24 years) transition by underlying long-term condition (LTC) groups and sex.
Marker size represents proportion of admissions during transition (age 16-18 years) with a record of LTCs (displayed on the right). Diamonds indicate group/overall estimates. IRR = incidence rate ratio, MEDReG disorders: Metabolic, Endocrine, Digestive, Renal or Genitourinary disorders

Figure 4: Incidence rate ratios for emergency admissions before (10-15 years) and after (19-24 years) transition by primary diagnosis and sex.
Marker size represents proportion of admissions at age 16-18 years with each primary diagnosis. The 15 primary diagnoses shown represent 76.9% and 77.7% of all primary diagnoses for females and males, respectively. LTCs are highlighted in bold. IRR = incidence rate ratio

*Diseases of male genital organs (ICD-10 codes N40-N51) include torsion of testis, and inflammatory diseases of the male genital organs such as orchitis and epididymitis.

Figure 5: Average length of stay per admission by patient age
Grey area indicates age range for transition

For peer review only

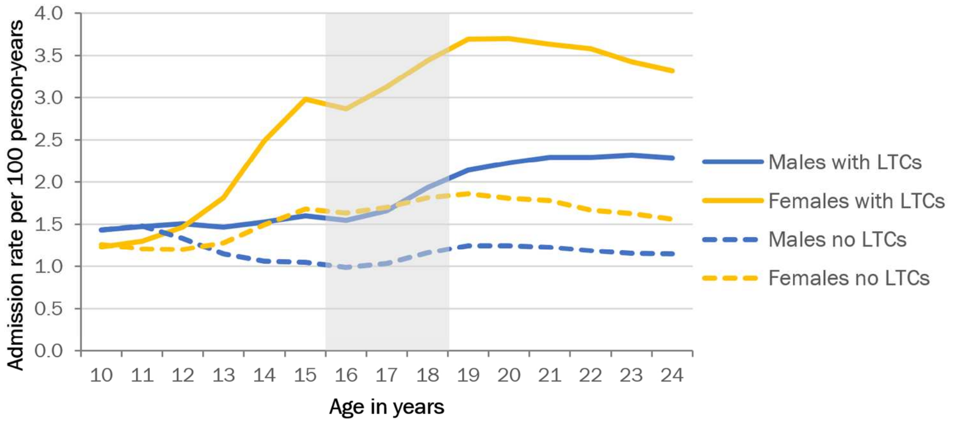

Emergency and elective admission rates by age and sex
LTCs = long-term conditions as measured in the index admission and admissions in the previous 5 years.
Grey area indicates age range for transition

206x90mm (300 x 300 DPI)

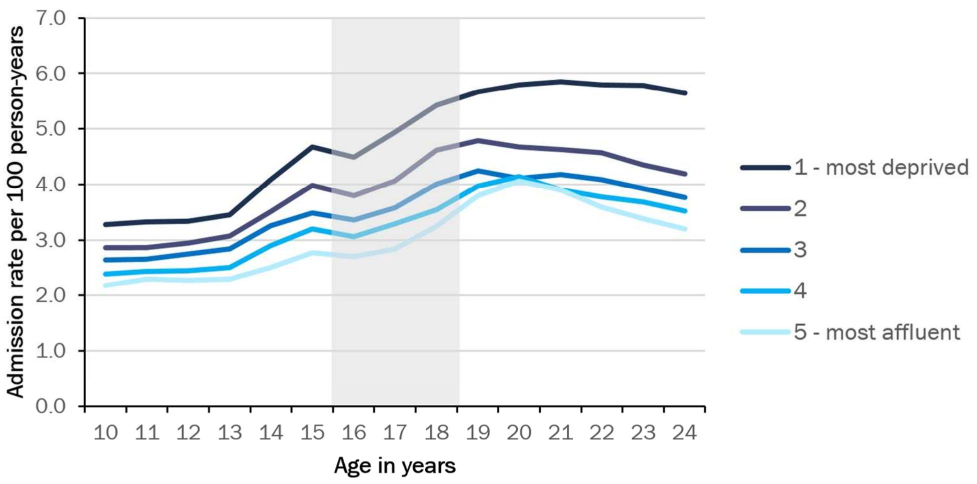

Emergency admissions by Index of Multiple Deprivation (IMD04) quintile
Grey area indicates age range for transition

185x90mm (300 x 300 DPI)

review only

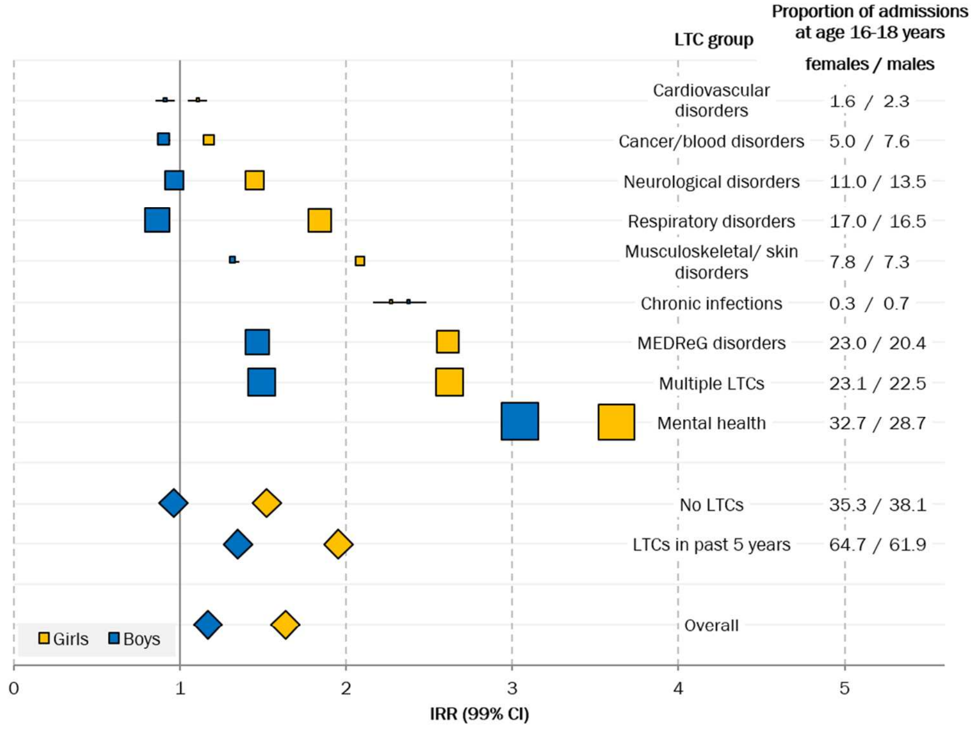

Incidence rate ratios for emergency admissions before (10-15 years) and after (19-24 years) transition by underlying long-term condition (LTC) groups and sex.
Marker size represents proportion of admissions during transition (age 16-18 years) with a record of LTCs (displayed on the right). Diamonds indicate group/overall estimates. IRR = incidence rate ratio, MEDReG disorders: Metabolic, Endocrine, Digestive, Renal or Genitourinary disorders

122x90mm (300 x 300 DPI)

For peer review only - http://bmjopen.bmj.com/site/about/guidelines.xhtml

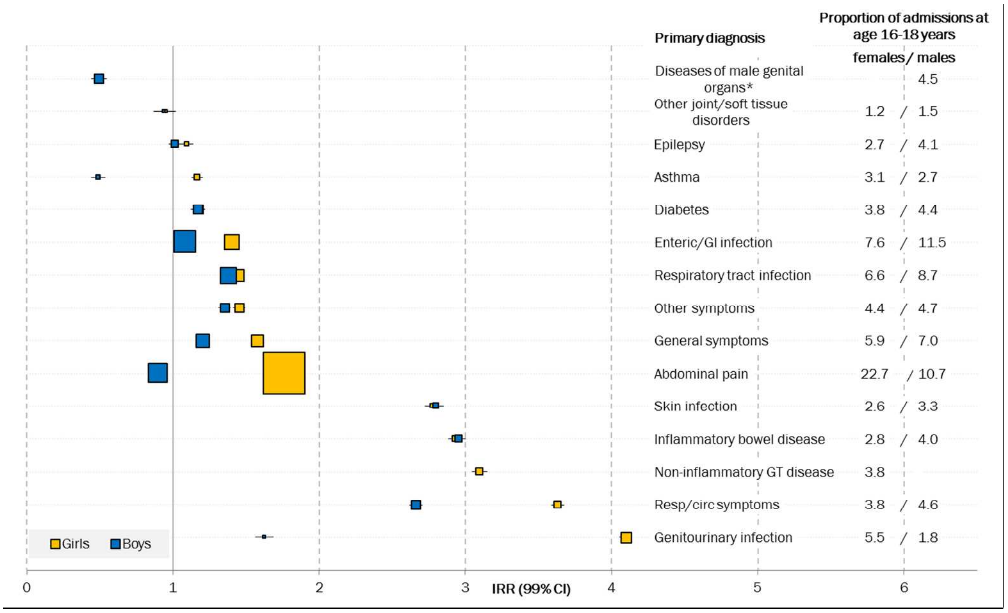

Incidence rate ratios for emergency admissions before (10-15 years) and after (19-24 years) transition by primary diagnosis and sex.
Marker size represents proportion of admissions at age 16-18 years with each primary diagnosis. The 15 primary diagnoses shown represent 76.9% and 77.7% of all primary diagnoses for females and males, respectively. LTCs are highlighted in bold. IRR = incidence rate ratio

*Diseases of male genital organs (ICD-10 codes N40-N51) include torsion of testis, and inflammatory diseases of the male genital organs such as orchitis and epididymitis.

148x90mm (300 x 300 DPI)

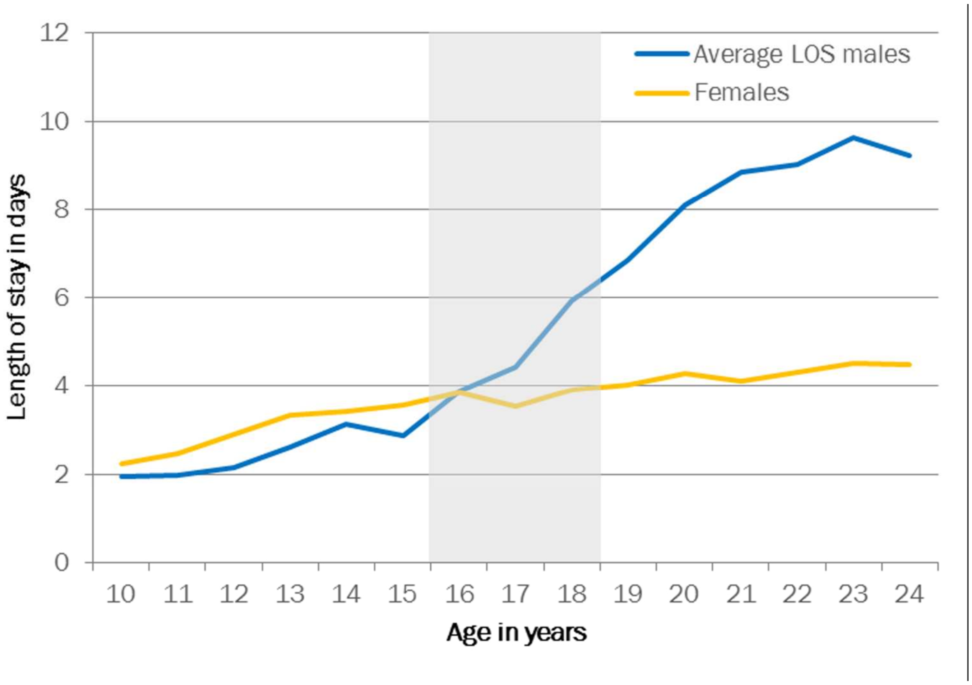

Average length of stay per admission by patient age
Grey area indicates age range for transition

133x90mm (300 x 300 DPI)

Appendix A: Code lists

In order to identify primary diagnoses groups in Table 2 and Figures in Appendix C, we used the following ICD-10 codes:

Table A1: ICD-10 codes used to define condition groups

Primary diagnoses group	ICD-10 codes
Long-term conditions	
Diabetes	E10-E14, G59.0, G63.2, I79.2, M14.2, N08.3, O24.0, O24.1, O24.2, O24.3, Y42.3
Asthma and chronic lower respiratory disease	J41-J47
Epilepsy	F80.3, G40.0, G40.1, G40.2, G40.3, G40.4, G40.6, G40.7, G41, G40.8, G40.9, R56.8, Y46.0, Y46.1, Y46.2, Y46.3, Y46.4, Y46.5, Y46.6
Non-infective enteritis and colitis / inflammatory bowel disease	K50, K51, K52
Mental health conditions	
Substance use	F10-F19
Schizophrenia, schizotypical and delusional disorders	F20-F29
Personality disorders	F60, F61, F69
Mood disorders	F30-F39
Infections	
Enteric / gastrointestinal infection	A00-A09, I88.0, K23.0, K23.1, K25-K28, K29.3, K29.4, K29.5, K35-K37, K52.8, K52.9, K61, K63.0, K63.2, K65.0, K67.8, K90.8, K93.0, R11
Respiratory infection	A15-A19, A48.1, A48.2, B59, J00-J06, J10-J22, J32, J36, J37, J39.0, J39.1, J40-J42, J43.0, J44.0, J47, J56, J85, J86, J98.8, N74.0, N74.1
Genitourinary infection	N30.0, N34.1, N35.1, N37, N39.0, N41.0, N41.1, N41.2, N41.3, N43.1, N45, N48.1, N48.2, N49, N51, N70-N74, N75.1, N76.4, N87
Symptoms and signs	
Abdominal pain	R10-R19, K59
General symptoms and signs	R50-R69
Respiratory and circulatory symptoms	R00-R09
Other symptoms	R20-49, R70-R99
Other conditions	
Non-inflammatory disease of the female genitourinary system	N80-N98
Other joint/soft tissue disorders	M20-M25, M70-M79
Diseases of male genital organs	N40-N51

For peer review only

Codes for long-term conditions (apart from sickle cell disease and inflammatory bowel disease) were taken from *Hardelid et al.*[1], codes for infections were taken from *Baker et al.*[2]. Other groups are based on ICD-10 subchapters.

1. Hardelid P, Dattani N, Davey J, et al. Overview of child deaths in the four UK countries. HQIP bit.ly/hardelidreport
2. Barker MG, Barnard LT, Kvalsvig A, et al. Increasing incidence of serious infectious diseases and inequalities in New Zealand: a national epidemiological study. *The Lancet* 2012;**379**:1112-9.

For peer review only

Appendix B: additional tables and figures

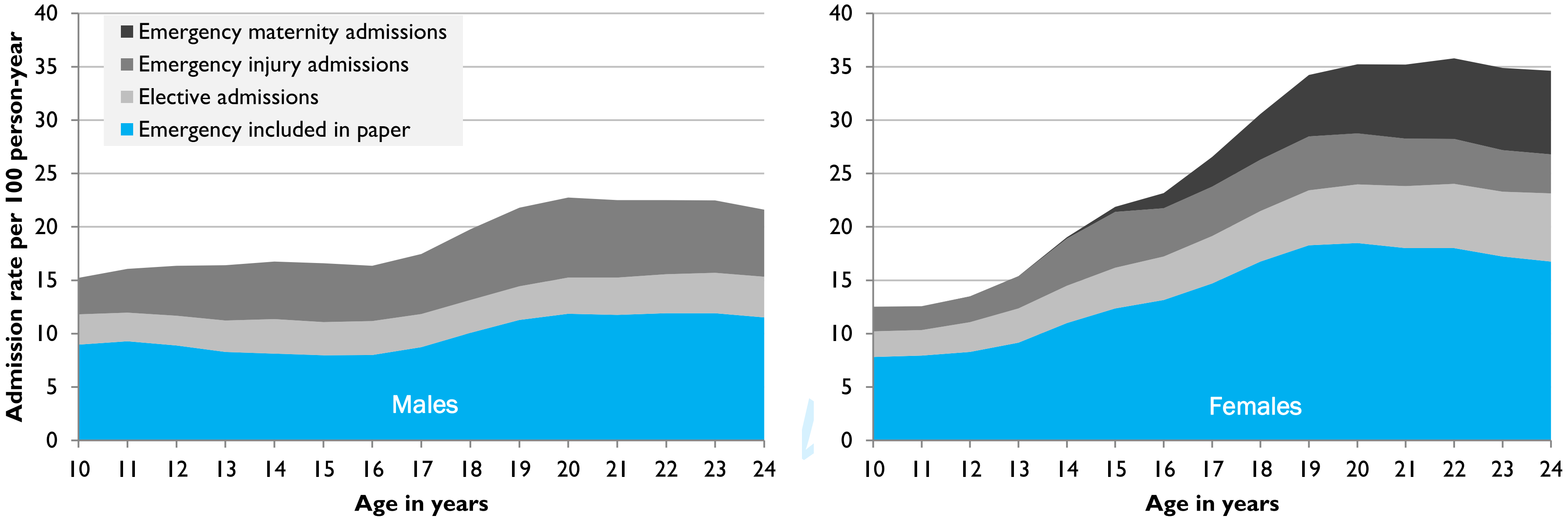

Figure B1: Emergency admission included in context: emergency admissions included in the primary analyses are shown in blue, while excluded admissions (elective admissions, emergency injury and maternity admissions) are shown in grey.

Table B1: Baseline characteristics of included admissions

	Overall	No LTC	LTC
**Covariate**	**Number of admissions (%)**		
**Total**	**1,109,978**	**410,144**	**699,834**
Before transition (10-15 years)	327,531 (29.5)	142,206 (34.7)	185,325 (26.5)
During transition (16-18 years)	224,722 (20.3)	81,676 (19.9)	143,046 (20.4)
After transition (19-24 years)	557,725 (50.3)	186,262 (45.4)	371,463 (53.1)
Females	644,994 (58.1)	229,651 (56.0)	415,343 (59.4)
IMD '04			
1 - Most deprived	319,837 (28.8)	108,211 (26.4)	211,626 (30.2)
2	242,428 (21.8)	85,930 (21.0)	156,498 (22.4)
3	198,237 (17.9)	74,583 (18.2)	123,690 (17.7)
4	172,479 (15.5)	68,364 (16.7)	104,115 (14.9)
5 – Least deprived	159,588 (14.4)	65,817 (16.1)	93,771 (13.4)
Missing	17,409 (1.6)	7,239 (1.8)	10,134 (1.5)
Ethnicity			
White	860,552 (77.5)	307,092 (74.9)	553,460 (79.1)
Black or Black British	45,564 (4.1)	14,093 (3.4)	31,471 (4.5)
Asian or Asian British	75,393 (6.8)	30,460 (7.4)	44,933 (6.4)
Mixed	44,863 (4.0)	17,414 (4.3)	27,449 (3.9)
Unknown	20,251 (1.8)	10,118 (2.5)	10,133 (1.5)
Missing	63,355 (5.7)	30,967 (7.6)	32,388 (4.6)

For peer review only

Table B2: Admission rates, incidence rate ratios and trends in rates before, during, and after transition to adult health care for children with and without underlying long-term conditions (LTCs)

	Before transition		After transition		Before	During	After
	Percentage of admissions 10-15 years	Average rate 10-15 years	Average rate 19-24 years		Trend age 10-15 years	Trend age 16-18 years	Trend age 19-25 years
**FEMALES**		Rate per 1,000 person-years		IRR (99% CI)†	Annual change in admission rate per 1,000 person-years		
**Overall***	**100**	**32.5**	**52.7**	**1.62 (1.61 – 1.63)**	**↑4.48**	**↑3.78**	**↓-1.39**
No LTCs	41.8	13.6	17.1	1.26 (1.25 – 1.28)	↗0.87	↗0.92	↘-0.61
History of LTCs†	58.2	18.9	35.5	1.88 (1.87 – 1.89)	↑3.61	↑2.86	↘-0.78
**LTC groups**							
Mental health disorders	20.5	6.7	17.8	2.67 (2.65 – 2.68)	↑2.87	↑1.48	↘-0.63
Substance use	2.9	0.9	4.6	4.94 (4.90 – 4.98)	↗0.51	↗0.77	→-0.09
Cancer/blood disorders	6.8	2.2	2.6	1.20 (1.17 – 1.23)	→0.09	→-0.02	→0.00
Chronic infections	0.4	0.1	0.3	2.34 (2.22 – 2.45)	→0.02	→0.02	→0.01
Respiratory disorders	16.3	5.3	10.3	1.94 (1.92 – 1.96)	↗0.53	↑1.11	↘-0.23
Asthma	11.4	3.7	6.6	1.79 (1.77 – 1.81)	↗0.29	↗0.79	↘-0.20
MEDReG‡ disorders	18.8	6.1	16.2	2.66 (2.64 – 2.67)	↗0.66	↑2.12	↗0.15
Inflammatory bowel disease	3.2	1.0	2.4	2.35 (2.31 – 2.39)	→0.05	↗0.30	→0.04
Diabetes	5.2	1.7	2.0	1.16 (1.13 – 1.20)	↗0.19	↗0.13	↘-0.15
Musculoskeletal/skin disorders	7.7	2.5	5.3	2.11 (2.09 – 2.14)	↗0.33	↗0.40	→0.05
Neurological disorders	13.1	4.3	6.5	1.52 (1.50 – 1.54)	↗0.44	↗0.23	→0.06
Epilepsy	5.2	1.7	1.9	1.13 (1.09 – 1.21)	↗0.11	→-0.02	→-0.04
Cardiovascular disorders	2.1	0.7	0.8	1.16 (1.10 – 1.21)	→0.05	→-0.07	→-0.01
Multiple LTCs	17.8	5.8	15.2	2.62 (2.61 – 2.64)	↑1.00	↑1.66	→-0.05
**MALES**					Annual change in admission rate per 1,000 person-years		
**Overall***	**100**	**27.5**	**34.6**	**1.26 (1.25 – 1.27)**	**↘-0.70**	**↑2.80**	**→0.07**
No LTCs	45.3	12.4	12.0	0.96 (0.95 – 0.98)	↘-0.97	↗0.88	↘-0.23

For peer review only

History of LTCs†	54.7	15.0	22.6	1.50 (1.49 – 1.51)	↗0.27	↑1.91	↗0.30
**LTC groups**							
Mental health disorders	13.4	3.7	12.6	3.43 (3.41 – 3.45)	↗0.61	↑1.75	↗0.30
Substance use	2.0	0.5	4.9	9.01 (8.97 – 9.06)	↗0.29	↗0.98	↗0.22
Cancer/blood disorders	8.8	2.4	2.2	0.92 (0.89 – 0.96)	→0.02	→-0.07	→0.02
Chronic infections	0.5	0.1	0.5	2.44 (2.34 – 2.55)	→0.01	→0.02	→0.05
Respiratory disorders	19.0	5.2	4.9	0.94 (0.92 – 0.96)	↘-0.33	↗0.17	→-0.08
Asthma	13.8	3.8	3.1	0.82 (0.79 – 0.84)	↘-0.36	↗0.14	→-0.09
MEDReG‡ disorders	19.1	5.2	7.9	1.51 (1.49 – 1.53)	↗0.11	↗0.54	↗0.28
Inflammatory bowel disease	4.2	1.1	1.9	1.62 (1.58 – 1.66)	→-0.03	↗0.18	→0.05
Diabetes	4.7	1.3	1.4	1.08 (1.04 – 1.12)	↗0.11	↗0.16	→-0.06
Musculoskeletal/skin disorders	6.8	1.9	2.6	1.38 (1.35 – 1.41)	→0.08	→0.08	→0.10
Neurological disorders	14.5	4.0	4.1	1.04 (1.02 – 1.06)	→0.00	→-0.03	→0.07
Epilepsy	5.9	1.6	1.8	1.14 (1.10 – 1.17)	→-0.04	→0.00	→0.02
Cardiovascular disorders	2.6	0.7	0.7	0.94 (0.88 – 0.99)	→-0.02	→-0.06	→0.02
Multiple LTCs	19.0	5.2	8.5	1.63 (1.61 – 1.64)	↗0.19	↗0.55	↗0.23

*Excluding admissions for injury or maternity; †At least 1 admission with a record of a long-term condition in the previous 5 years (including current admission); ‡Metabolic, endrocrine, digestive, renal or genitourinary disorders

†Rate ratio comparing emergency admission rates before and after transition

Trends:

↑ increasing by ≥1.0 admission per 1,000 person-year (py);

↗ increasing by 0.1-0.99 per 1,000py;

→ stable at -0.1 to 0.1 per 1,000 py;

↘ decreasing by -0.1 to -0.99 per 1,000py;

↓ decreasing by ≤-1.0 per 1,000py

For peer review only

Table B3: Admission rates, incidence rate ratios and trends in rates before, during, and after transition to adult health care by primary diagnosis

	Proportion of admissions 10-15 years	Average rate 10-15 years	Average rate 19-24 years	IRR (99% CI)†	Trend age 10-15 years	Trend age 16-18 years	Trend age 19-25 years
**FEMALES***	Rate per 1,000 person-years				Annual change in admission rate per 1,000 person-years		
**Long-term conditions**							
Any long-term condition	26.4	9.8	19.8	2.02 (2.00 – 2.03)	↑1.04	↑2.54	↘-0.12
Diabetes	3.7	1.2	1.4	1.18 (1.13 – 1.22)	→0.10	↗0.26	↘-0.22
Asthma	4.5	1.4	1.6	1.17 (1.13 – 1.20)	↘-0.11	↗0.14	→-0.02
Inflammatory bowel disease	2.3	0.7	2.1	2.93 (2.88 – 2.98)	→0.03	↗0.37	→0.03
Epilepsy	3.4	1.1	1.2	1.09 (1.05 – 1.14)	→0.07	→0.02	→-0.06
**Infection**							
Any infection	25.4	8.4	14.4	1.72 (1.70 – 1.73)	↗0.26	↑1.66	↘-0.50
Enteric/GI infection	9.4	2.9	4.1	1.40 (1.38 – 1.43)	→0.06	↗0.28	↘-0.11
Respiratory tract infection	7.0	2.2	3.1	1.45 (1.42 – 1.48)	→0.05	↗0.30	↘-0.13
Genitourinary infection	2.5	0.8	3.2	4.10 (4.05 – 4.14)	↗0.11	↗0.58	↘-0.14
**Symptoms**							
Any symptoms	35.8	11.3	20.5	1.83 (1.81 – 1.84)	↑1.31	↑1.95	↘-0.48
Abdominal pain	22.6	7.1	12.5	1.76 (1.75 – 1.78)	↗0.79	↑1.26	↘-0.46
General symptoms	6.9	2.2	3.4	1.58 (1.55 – 1.61)	↗0.31	↗0.12	→-0.04
**Other**							
Any other	12.4	2.6	4.9	1.87 (1.85 – 1.90)	↗0.33	↗0.44	→0.06
Non-inflammatory disease of the female GT	2.3	0.6	1.8	3.10 (3.04 – 3.15)	↗0.22	↗0.20	→-0.04
Other joint/soft tissue disorders	2.4	0.5	0.5	0.95 (0.89 – 1.02)	→-0.01	→0.04	→0.00
**MALES***					Annual change in admission rate per 1,000 person-years		

For peer review only

**Long-term conditions**							
Any long-term condition	25.8	8.9	14.4	1.61 (1.60 – 1.62)	↗0.17	↑1.50	↗0.29
Diabetes	3.3	0.9	1.1	1.17 (1.12 – 1.22)	→0.09	↗0.21	↘-0.13
Asthma	5.5	1.5	0.8	0.49 (0.44 – 0.53)	↘-0.29	→-0.05	→-0.03
Inflammatory bowel disease	2.9	0.7	2.0	2.95 (2.91 – 3.00)	→0.02	↗0.24	→0.01
Epilepsy	4.1	1.2	1.2	1.01 (0.97 – 1.06)	→-0.01	→0.05	→-0.01
**Infection**							
Any infection	28.6	8.5	10.9	1.29 (1.27 – 1.30)	↘-0.47	↑1.08	↘-0.14
Enteric/GI infection	12.7	3.6	3.9	1.08 (1.06 – 1.11)	↘-0.15	↗0.15	→-0.06
Respiratory tract infection	7.0	2.0	2.7	1.38 (1.35 – 1.41)	↘-0.13	↗0.42	↘-0.11
Genitourinary infection	1.7	0.5	0.8	1.63 (1.57 – 1.69)	→-0.03	↗0.11	→-0.01
**Symptoms**							
Any symptoms	29.2	8.3	10.1	1.21 (1.20 – 1.23)	↘-0.52	↗0.97	→0.05
Abdominal pain	15.4	4.4	4.0	0.90 (0.88 – 0.92)	↘-0.54	↗0.28	→-0.02
General symptoms	6.8	1.9	2.3	1.20 (1.17 – 1.24)	→0.02	↗0.19	→-0.01
**Other**							
Any other	16.4	3.4	3.7	1.10 (1.08 – 1.13)	↘-0.21	↗0.26	→0.02
Diseases of male genital organs	6.0	1.2	0.6	0.49 (0.44 – 0.55)	→-0.08	→-0.03	→-0.04
Other joint/soft tissue disorders	2.3	0.5	0.4	0.94 (0.87 – 1.01)	→-0.06	→0.00	→0.02

*Excluding admissions for injury or maternity

†Incidence rate ratio comparing emergency admission rates before and after transition

Trends:

↑ increasing by ≥1.0 admission per 1,000 person-year (py);

↗ increasing by 0.1-0.99 per 1,000py;

→ stable at -0.1 to 0.1 per 1,000 py;

↘ decreasing by -0.1 to -0.99 per 1,000py;

↓ decreasing by ≤-1.0 per 1,000py

For peer review only

Table B4: Most frequent diagnoses for CYP with and without underlying LTCs

Rank	CYP with LTCs 10-15 years	CYP with LTCs 16-18 years	CYP with LTCs 19-24 years	CYP without LTCs 10-15 years	CYP without LTCs 16-18 years	CYP without LTCs 19-24 years
	Diagnosis group* (frequency, % of admissions)			Diagnosis group* (frequency, % of admissions)		
1	Asthma (13,209, 7.1%)	Abdominal and pelvic pain (14,643, 10.2%)	Abdominal and pelvic pain (41,809, 11.3%)	Abdominal and pelvic pain (34,348, 24.2%)	Abdominal and pelvic pain (19,231, 23.6%)	Abdominal and pelvic pain (39,952, 21.5%)
2	Abdominal and pelvic pain (13,015, 7.0%)	Poisoning by nonopioid analgesics, antipyretics and antirheumatics (12,835, 9.0%)	Poisoning by nonopioid analgesics, antipyretics and antirheumatics (20,632, 5.6%)	Acute appendicitis (11,699, 8.2%)	Acute appendicitis (6,266, 7.7%)	Pain in throat and chest (10,574, 5.7%)
3	Insulin-dependent diabetes mellitus (10,277, 5.6%)	Insulin-dependent diabetes mellitus (6,694, 4.7%)	Pain in throat and chest (10,737, 2.9%)	Other functional intestinal disorders (4,783, 3.4%)	Acute tonsillitis (4,272, 5.2%)	Acute appendicitis (10,206, 5.5%)
4	Poisoning by nonopioid analgesics, antipyretics and antirheumatics (10,105, 5.5%)	Asthma (4,191, 2.9%)	Other noninfective gastroenteritis and colitis (10,546, 2.8%)	Viral infection of unspecified site (4,615, 3.3%)	Pain in throat and chest (3,105, 3.8%)	Headache (6,984, 3.8%)
5	Epilepsy (6,146, 3.3%)	Mental and behavioral disorders due to use of alcohol (3,796, 2.7%)	Insulin-dependent diabetes mellitus (9,971, 2.7%)	Acute tonsillitis (4,275, 3.0%)	Other disorders of urinary system (3,046, 3.7%)	Other disorders of urinary system (6,029, 3.2%)

*Diagnoses are grouped by 3-character ICD codes (e.g. E84 for cystic fibrosis)
Signs and symptoms are highlighted

Table B5: Most frequent diagnoses for long (10+ days) lengths of stay by age group and gender

Rank	Males 10-15 years	Males 16-18 years	Males 19-24 years	Females 10-15 years	Females 16-18 years	Females 19-24 years
	Diagnosis group* (frequency, *average length of stay*)			Diagnosis group* (frequency, *average length of stay*)		
1	Cystic Fibrosis (414, *8.6*)	Unknown and unspecified causes of morbidity (354, ***53.4***)	Schizophrenia (3,268, ***140.0***)	Cystic fibrosis (612, *8.6*)	Cystic fibrosis (390, *8.8*)	Abdominal and pelvic pain (1,111, *2.0*)
2	Pneumonia, organism unspecified (205, *4.6*)	Pneumothorax (313, *6.0*)	Unknown and unspecified causes of morbidity (2,196, ***97.6***)	Eating disorders (519, ***41.9***)	Abdominal and pelvic pain (386, *1.8*)	Unknown and unspecified causes of morbidity (1,096, ***40.5***)
3	Lymphoid leukaemia (193, *4.9*)	Schizophrenia (268, ***119.7***)	Acute and transient psychotic disorders (773, ***44.3***)	Pneumonia, organism unspecified (221, *4.6*)	Unknown and unspecified causes of morbidity (328, ***53.4***)	Specific personality disorders (917, ***32.5***)
4	Osteomyelitis (175, ***11.0***)	Cystic Fibrosis (254, *8.8*)	Crohn disease (648, *7.4*)	Lymphoid leukaemia (164, *4.9*)	Eating disorders (316, ***49.5***)	Cystic fibrosis (761, ***11.4***)
5	Unspecified acute lower respiratory infection / Acute appendicitis (171, *3.2 / 3.1*)	Ulcerative colitis (240, *8.9*)	Ulcerative colitis (590, *8.7*)	Unspecified acute lower respiratory infection (160, *3.2*)	Crohn disease (211, *7.0*)	Crohn disease (721, *7.4*)

*Diagnoses are grouped by 3-character ICD codes (e.g. E84 for cystic fibrosis)
Average lengths of stay are calculated for all emergency admissions (excluding injury and maternity-related admissions). Average lengths of stay of 10 days or greater are shown in bold.
Mental health conditions are highlighted in blue, unspecified conditions and conditions that could represent psychosomatic symptoms are highlighted in yellow.

For peer review only

Appendix C: Sensitivity analyses

In Figure A1 in Appendix A, we showed the overall admission rate for children and young people aged 10-24 years old. Here we show the results, as they would have been in Table 1, if we had included all emergency admission, compared to the results presented in the paper (excluding injury admissions, and maternity/pregnancy related emergency admissions for females, Table C1).

Table C1: Sensitivity analysis of exclusion criteria on rates and trends in emergency admission rates before, during and transition from pediatric to adult care

	Proportion of admissions 10-15 years	Average rate 10-15 years	Average rate 19-24 years		Trend age 10-15 years	Trend age 16-18 years	Trend age 19-25 years
**FEMALES**	Rate per 1,000 person-years			IRR† (99% CI)	Annual change in admission rate per 1,000 py		
Overall	*100*	39.4	81.6	2.07 (2.06 – 2.08)	↑3.61	↑2.86	↘-0.78
Overall, excluding injury/maternity admissions	82.4	32.5	52.7	1.62 (1.61 – 1.63)	↑4.48	↑3.78	↓-1.39
Injury admissions	16.7	6.6	6.9	1.05 (1.03-1.06)	→0.10	↗0.41	↘-0.31
Maternity/pregnancy admissions	0.9	0.4	22.2	61.9 (61.8 – 62.0	↗0.26	↑4.62	↑1.34
**MALES**							
Overall	*100*	41.0	50.8	1.24 (1.23 – 1.25)	↗0.27	↑1.91	↗0.30
Overall, excluding injury admissions	67.0	27.5	34.6	1.26 (1.25 – 1.27)	↘-0.70	↑2.80	→0.07
Injury admissions	33.0	13.5	16.2	1.20 (1.19 – 1.21)	↗0.94	↑1.49	↘-0.52

*Excluding maternity/pregnancy related admissions and injury admissions
†Incidence rate ratio comparing emergency admission rates before and after transition

By excluding injury and maternity/pregnancy related emergency admissions, we have slightly underestimated the increase in emergency admission rates across transition for both males and females.

For peer review only

In the manuscript, we assessed children with underlying long-term conditions as a separate group. We assessed whether children and young people had a code indicating a LTC recorded in any admission record (emergency or elective) in the previous 5 years. As a sensitivity analysis, we explored the effects of only using admission records from the last year or last 3 years (Table C2).

Table C2: Sensitivity analysis of the effect of including long-term conditions (LTCs) recorded in the last year, last 3 years, or last 5 years

	Proportion of admissions 10-15 years	Average rate 10-15 years	Average rate 19-24 years		Trend age 10-15 years	Trend age 16-18 years	Trend age 19-25 years
**FEMALES***	Rate per 1,000 person-years			IRR† (99% CI)	Annual change in admission rate per 1,000 py		
No LTCs	41.8	13.6	17.1	1.26 (1.25 – 1.28)	↗0.87	↗0.92	↘-0.61
LTC recorded in previous year	56.0	18.2	33.3	1.83 (1.82-1.84)	↑3.49	↑2.54	↘-0.77
LTC recorded in previous 3 years	57.5	18.7	34.7	1.86 (1.85-1.87)	↑3.58	↑2.71	↘-0.79
LTC recorded in previous 5 years	58.2	18.9	35.5	1.88 (1.87-1.89)	↑3.61	↑2.86	↘-0.78
**MALES***							
No LTCs	45.3	12.4	12.0	0.96 (0.95-0.98)	↘-0.97	↗0.88	↘-0.23
LTC recorded in previous year	52.6	14.4	21.7	1.50 (1.49 – 1.51)	↗0.30	↑1.84	↗0.26
LTC recorded in previous 3 years	55.8	15.3	22.3	1.45 (1.44 – 1.47)	↗0.29	↑1.90	↗0.28
LTC recorded in previous 5 years	56.0	15.4	22.6	1.47 (1.46 – 1.48)	↗0.27	↑1.91	↗0.30

*Excluding maternity/pregnancy related admissions and injury admissions
†Incidence rate ratio comparing emergency admission rates before and after transition

When using records for the previous five years, we find slightly higher emergency admission rates for children with underlying long-term conditions compared to looking back just one or three years. However, the relatively small differences indicate most LTCs were recorded in the year prior to the index emergency admission.

For peer review only

STROBE 2007 (v4) Statement—Checklist of items that should be included in reports of *cross-sectional studies*

Section/Topic	Item #	Recommendation	Reported on page #
**Title and abstract**	1	(*a*) Indicate the study's design with a commonly used term in the title or the abstract	1
		(*b*) Provide in the abstract an informative and balanced summary of what was done and what was found	2
**Introduction**			
Background/rationale	2	Explain the scientific background and rationale for the investigation being reported	3-4
Objectives	3	State specific objectives, including any prespecified hypotheses	4
**Methods**			
Study design	4	Present key elements of study design early in the paper	5
Setting	5	Describe the setting, locations, and relevant dates, including periods of recruitment, exposure, follow-up, and data collection	
Participants	6	(*a*) Give the eligibility criteria, and the sources and methods of selection of participants	5
Variables	7	Clearly define all outcomes, exposures, predictors, potential confounders, and effect modifiers. Give diagnostic criteria, if applicable	6
Data sources/ measurement	8*	For each variable of interest, give sources of data and details of methods of assessment (measurement). Describe comparability of assessment methods if there is more than one group	5-6
Bias	9	Describe any efforts to address potential sources of bias	6-7
Study size	10	Explain how the study size was arrived at	5
Quantitative variables	11	Explain how quantitative variables were handled in the analyses. If applicable, describe which groupings were chosen and why	6-7
Statistical methods	12	(*a*) Describe all statistical methods, including those used to control for confounding	6-7
		(*b*) Describe any methods used to examine subgroups and interactions	7
		(*c*) Explain how missing data were addressed	7
		(*d*) If applicable, describe analytical methods taking account of sampling strategy	n/a
		(*e*) Describe any sensitivity analyses	7
**Results**			

For peer review only

Participants	13*	(a) Report numbers of individuals at each stage of study—eg numbers potentially eligible, examined for eligibility, confirmed eligible, included in the study, completing follow-up, and analysed	7
		(b) Give reasons for non-participation at each stage	n/a
		(c) Consider use of a flow diagram	n/a
Descriptive data	14*	(a) Give characteristics of study participants (eg demographic, clinical, social) and information on exposures and potential confounders	7-8 (+ appendix A)
		(b) Indicate number of participants with missing data for each variable of interest	7-8
Outcome data	15*	Report numbers of outcome events or summary measures	7-8
Main results	16	(*a*) Give unadjusted estimates and, if applicable, confounder-adjusted estimates and their precision (eg, 95% confidence interval). Make clear which confounders were adjusted for and why they were included	8-10
		(*b*) Report category boundaries when continuous variables were categorized	9-10
		(*c*) If relevant, consider translating estimates of relative risk into absolute risk for a meaningful time period	10
Other analyses	17	Report other analyses done—eg analyses of subgroups and interactions, and sensitivity analyses	10-11 (+ appendix C)
**Discussion**			
Key results	18	Summarise key results with reference to study objectives	11
Limitations	19	Discuss limitations of the study, taking into account sources of potential bias or imprecision. Discuss both direction and magnitude of any potential bias	12-13
Interpretation	20	Give a cautious overall interpretation of results considering objectives, limitations, multiplicity of analyses, results from similar studies, and other relevant evidence	13-14
Generalisability	21	Discuss the generalisability (external validity) of the study results	11-12
**Other information**			
Funding	22	Give the source of funding and the role of the funders for the present study and, if applicable, for the original study on which the present article is based	16

*Give information separately for cases and controls in case-control studies and, if applicable, for exposed and unexposed groups in cohort and cross-sectional studies.

Note: An Explanation and Elaboration article discusses each checklist item and gives methodological background and published examples of transparent reporting. The STROBE checklist is best used in conjunction with this article (freely available on the Web sites of PLoS Medicine at http://www.plosmedicine.org/, Annals of Internal Medicine at http://www.annals.org/, and Epidemiology at http://www.epidem.com/). Information on the STROBE Initiative is available at www.strobe-statement.org.

For peer review only
